# The innate immune regulator MyD88 dampens fibrosis during zebrafish heart regeneration

**DOI:** 10.1038/s44161-024-00538-5

**Published:** 2024-09-13

**Authors:** Pinelopi Goumenaki, Stefan Günther, Khrievono Kikhi, Mario Looso, Rubén Marín-Juez, Didier Y. R. Stainier

**Affiliations:** 1 Department of Developmental Genetics, Max Planck Institute for Heart and Lung Research, Bad Nauheim, Germany; 2 DZHK German Centre for Cardiovascular Research, Partner Site Rhine-Main, Bad Nauheim, Germany; 3 Cardio-Pulmonary Institute (CPI), Bad Nauheim, Germany; 4 Bioinformatics and Deep Sequencing Platform, Max Planck Institute for Heart and Lung Research, Bad Nauheim, Germany; 5 Flow Cytometry Service Group, Max Planck Institute for Heart and Lung Research, Bad Nauheim, Germany; 6 Bioinformatics Core Unit (BCU), Max Planck Institute for Heart and Lung Research, Bad Nauheim, Germany; 7 Centre Hospitalier Universitaire Sainte-Justine Research Centre, Montreal, Quebec Canada; 8 Department of Pathology and Cell Biology, University of Montreal, Montreal, Quebec Canada

**Keywords:** Innate immunity, Cardiac regeneration, Cardiovascular genetics, Transcriptomics

## Abstract

The innate immune response is triggered rapidly after injury and its spatiotemporal dynamics are critical for regeneration; however, many questions remain about its exact role. Here we show that MyD88, a key component of the innate immune response, controls not only the inflammatory but also the fibrotic response during zebrafish cardiac regeneration. We find in cryoinjured *myd88*^−/−^ ventricles a significant reduction in neutrophil and macrophage numbers and the expansion of a collagen-rich endocardial population. Further analyses reveal compromised PI3K/AKT pathway activation in the *myd88*^−/−^ endocardium and increased myofibroblasts and scarring. Notably, endothelial-specific overexpression of *myd88* reverses these neutrophil, fibrotic and scarring phenotypes. Mechanistically, we identify the endocardial-derived chemokine gene *cxcl18b* as a target of the MyD88 signaling pathway, and using loss-of-function and gain-of-function tools, we show that it controls neutrophil recruitment. Altogether, these findings shed light on the pivotal role of MyD88 in modulating inflammation and fibrosis during tissue regeneration.

## Abstract (web summary)

Goumenaki et al. uncover that during zebrafish cardiac regeneration, MyD88 signaling promotes the inflammatory response to injury and attenuates the endocardial-mediated fibrotic response.

## Main

Myocardial infarction is the most common cause of cardiac injury in humans, often leading to an irreversible loss of heart muscle tissue^1,2^. Unlike the adult mammalian heart, the zebrafish heart can regenerate lost tissue upon different types of injuries, making it a powerful animal model to study heart regeneration^3–6^.

Immediately after cardiac injury, tissue damage triggers the activation of a sterile inflammatory response, with immune cells infiltrating the injured area, clearing dead cells and debris, modifying the extracellular matrix (ECM) and initiating signaling cascades^7,8^. As shown previously, this response is programmed to be resolved timely and is essential for triggering the regenerative response in both zebrafish^9–11^ and neonatal mice^12^. On the other hand, in the non-regenerative adult mammalian heart, inflammation persists longer and has been linked with tissue damage after myocardial infarction^13,14^. Thus, it is essential that the inflammatory response is tightly controlled to allow for successful regeneration^14^.

Cardiac inflammation after injury involves the release of damage-associated molecular pattern (DAMP) molecules from dying and stressed cells, which bind to Toll-like receptors (TLRs)^15–17^. Importantly, most TLRs, as well as interleukin-1 receptors (IL-1Rs), signal through the same adaptor molecule, myeloid differentiation factor 88 (MyD88)^18,19^. While the MyD88 signaling pathway is involved in the activation of the immune response, a comprehensive understanding of its role in cardiac repair and regeneration remains elusive, with studies reporting conflicting results^14,20,21^. As excessive and persistent inflammation has been linked with adverse regeneration outcomes, studies have often focused on blocking MyD88 or TLR function to restrict inflammation. Limiting inflammation in MyD88 or TLR loss-of-function (LOF) mouse models improved their regenerative potential^22–24^. Conversely, TLR4-MyD88 activation in mesenchymal stem cells conferred cardioprotective effects in cardiac ischemia–reperfusion injury models in mouse^25^, TLR4-MyD88 activation in cardiomyocytes (CMs) reduced their apoptosis in vitro^26^, and MyD88 cardioprotective effects were highlighted in a rat aortic banding model^27^. The critical role of MyD88 signaling in preserving cardiac function and limiting progression to heart failure was also shown in a dominant negative MyD88 transgenic mouse model^28^. Depletion of MyD88 in T cells results in increased inflammation and fibrosis after transverse aortic constriction in mouse^29^. While most of these studies were carried out in non-regenerative mammalian models, understanding the role of MyD88 in a regenerative system should provide insights into the pathogenesis of cardiovascular diseases and the development of new treatment approaches. In addition, the precise roles of the MyD88 signaling axis beyond cardiovascular inflammation and its contribution to other responses necessary for regeneration have so far been largely overlooked. Therefore, we directed our study toward unraveling the cell-specific functions of MyD88 signaling during cardiac regeneration in zebrafish.

In this study, we use newly generated genetic tools and transcriptomic profiling to investigate the role of MyD88 signaling during zebrafish cardiac regeneration at several stages and across different cell types; we find that MyD88 not only has a pivotal role in the immune response, but also a previously unidentified function in limiting endothelial-mediated fibrosis. Specifically, we show that in addition to its role in neutrophil and macrophage recruitment, MyD88 is important to limit a fibrotic response by regulating the transcriptome of endocardial and mesenchymal cells and by limiting myofibroblast numbers, fibrin levels and scar sizes. Furthermore, we identify a critical role for MyD88 signaling in regulating the phosphoinositide 3 kinase (PI3K)/AKT pathway in the injured endocardium. Notably, using an endothelial-specific mutant rescue strategy, we reveal the essential role of MyD88 in endothelial cells to promote neutrophil recruitment and limit the fibrotic response. Mechanistically, we identify the endocardial chemokine gene *cxcl18b* as a transcriptional target of the MyD88 signaling pathway and showed that it controls neutrophil recruitment.

## Results

### Reduced inflammatory cell numbers in injured *myd88*^−/−^ ventricles

**Fig. 1:**
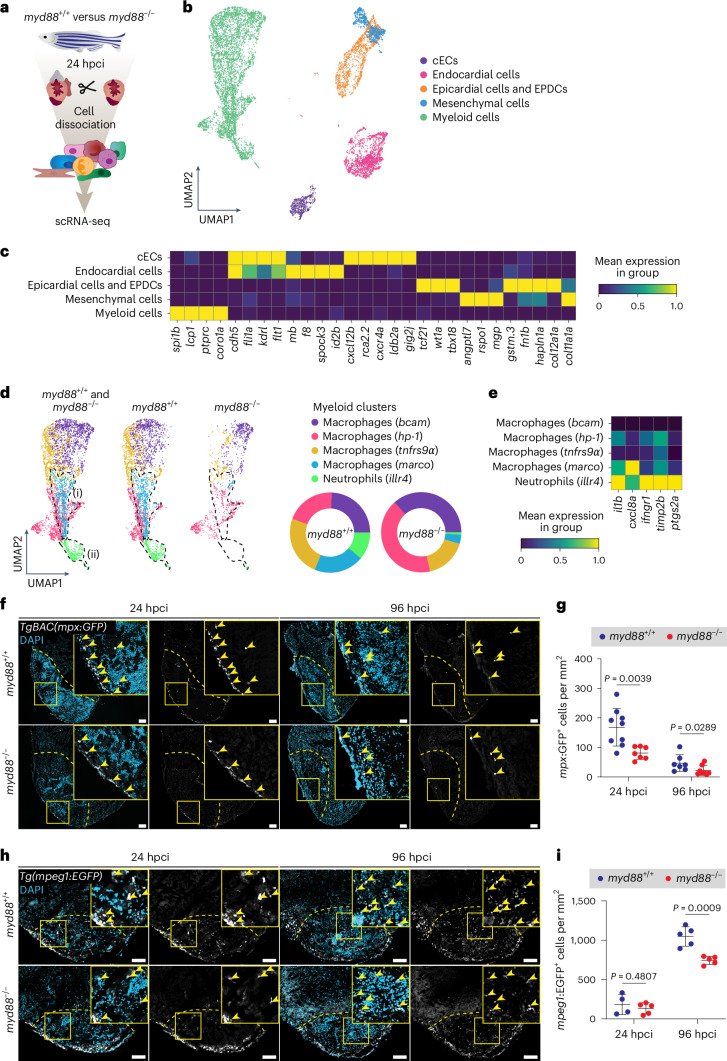
Reduced numbers of pro-inflammatory cells in cryoinjured *myd88*^−/−^ ventricles. **a**, Experimental plan for the scRNA-seq analysis performed in cryoinjured *myd88*^+/+^ and *myd88*^−/−^ ventricles at 24 hpci. **b**, Uniform manifold approximation and projection (UMAP) representation of the scRNA-seq clustering results. **c**, Heatmap showing the expression levels of the gene markers for the different cell types. **d**, UMAP representation of the myeloid subclusters from the scRNA-seq analysis. Areas (i) and (ii) enclose myeloid populations reduced in *myd88*^−/−^ ventricles. The pie charts show the proportions of different myeloid subclusters. **e**, Heatmap showing inflammatory gene (*il1b*, *cxcl8a*, *ifngr1*, *timp2b*, *ptgs2a*) expression levels in the myeloid subclusters. **f**, Representative images of GFP (neutrophils, white), with DAPI (DNA marker, blue) counterstaining on sections of cryoinjured *TgBAC(mpx:GFP)*; *myd88*^+/+^ and *TgBAC(mpx:GFP)*; *myd88*^−/−^ ventricles at 24 and 96 hpci. GFP; immunostaining for green fluorescent protein. **g**, *mpx:*GFP^+^ cell numbers in *myd88*^+/+^ and *myd88*^−/−^ injured tissues and border zone areas (100 μm) at 24 and 96 hpci. The dots in the graph represent individual ventricles; data are shown as the mean ± s.d.; *n* = 9 *myd88*^+/+^ and *n* = 7 *myd88*^−/−^ for 24 hpci; *n* = 7 *myd88*^+/+^ and *n* = 8 *myd88*^−/−^ for 96 hpci. Statistical tests: Student’s *t*-test for 24 hpci and Mann–Whitney *U*-test for 96 hpci. **h**, Representative images of immunostaining for EGFP (macrophages, white) with DAPI (DNA marker, blue) counterstaining on sections of cryoinjured *Tg(mpeg1:EGFP); myd88*^+/+^ and *Tg(mpeg1:EGFP); myd88*^−/−^ ventricles at 24 and 96 hpci. **i,** *mpeg1:*EGFP^*+*^ cell numbers in *myd88*^+/+^ and *myd88*^−/−^ injured tissues and border zone areas (100 μm) at 24 and 96 hpci. The dots in the graph represent individual ventricles; data are shown as the mean ± s.d.; *n* = 4 *myd88*^+/+^ and *n* = 5 *myd88*^−/−^ for 24 hpci; *n* = 5 *myd88*^+/+^ and *n* = 5 *myd88*^−/−^ for 96 hpci. Statistical tests: Student’s *t*-test. The yellow dashed lines delineate the injured area; the yellow arrowheads point to *mpx:*GFP^+^ (**f**) and *mpeg1:*EGFP^+^ (**h**) cells. Scale bars, 100 μm. Source data

**Extended Data Fig. 1:**
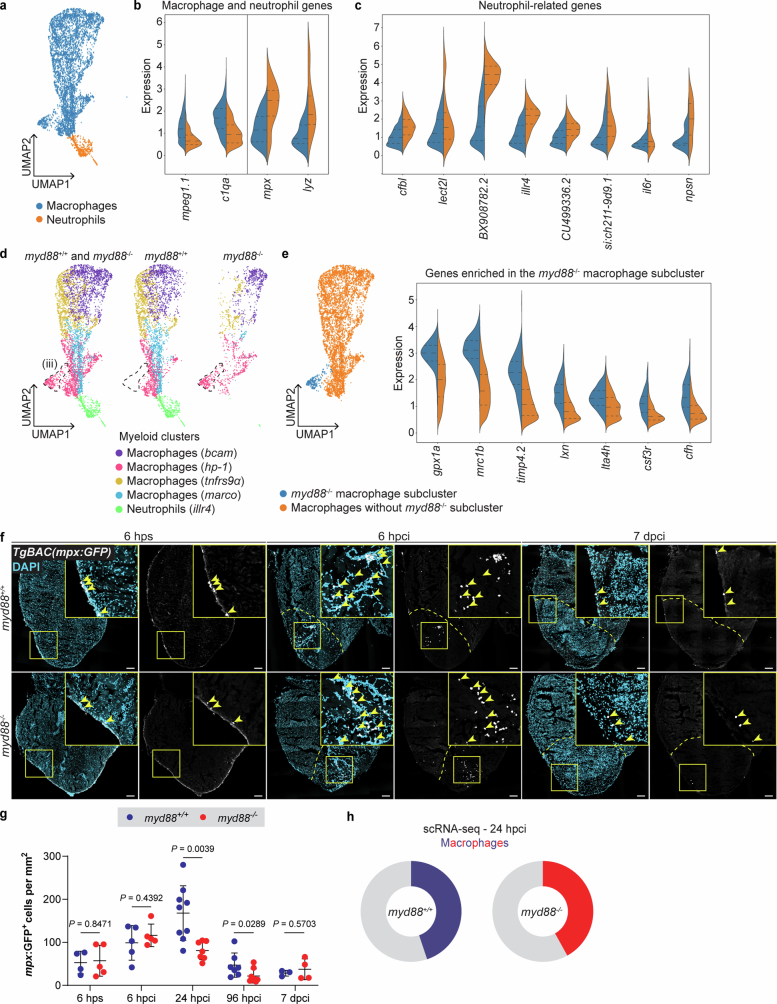
Neutrophils and macrophages are affected in cryoinjured *myd88*^*−/−*^ ventricles. **a**, UMAP representation of the macrophage and neutrophil clusters from the scRNA-seq analysis. **b**, Violin plots showing expression of known macrophage and neutrophil gene markers. **c**, Violin plots showing expression of neutrophil-enriched gene markers in the macrophage and neutrophil clusters. Genes selected from published datasets^34–36^. **d**, UMAP representation of the myeloid subclusters from the scRNA-seq analysis. Area (iii) corresponds to a macrophage subcluster that is increased in cryoinjured *myd88*^*−/−*^ ventricles. **e**, UMAP representation of this *myd88*^*−/−*^ macrophage subcluster (blue) and the other macrophages (orange), and violin plots showing enriched genes in the *myd88*^*−/−*^ macrophage subcluster (iii). **f**, Representative images of immunostaining for GFP (neutrophils, white) with DAPI (DNA marker, blue) counterstaining on sections of *TgBAC(mpx:GFP)*; *myd88*^+/+^ and *TgBAC(mpx*:*GFP)*; *myd88*^*−/−*^ ventricles at 6 hps, 6 hpci and 7 dpci. **g**, *mpx:*GFP^+^ cell numbers in *TgBAC(mpx:GFP)*; *myd88*^+/+^ and *TgBAC(mpx*:*GFP)*; *myd88*^*−/−*^ ventricles at 6 hps and injured tissues and border zone areas (100 μm) at 6, 24 and 96 hpci, as well as 7 dpci. The dots in the graph represent individual ventricles; data are shown as the mean ± s.d.; n = 4 *myd88*^+/+^, n = 5 *myd88*^*−/−*^ for 6 hps, n = 5 *myd88*^+/+^, n = 5 *myd88*^*−/−*^ for 6 hpci, n = 9 *myd88*^+/+^, n = 7 *myd88*^*−/−*^ for 24 hpci, n = 7 *myd88*^+/+^, n = 8 *myd88*^*−/−*^ for 96 hpci, and n = 3 *myd88*^+/+^, n = 4 *myd88*^*−/−*^ for 7 dpci. Statistical tests: Student’s *t*-test for 6 hps, 6, 24 hpci and 7 dpci, and Mann-Whitney *U*-test for 96 hpci. **h**, The pie charts show the proportion of macrophages in the scRNA-seq dataset at 24 hpci. Yellow dashed lines delineate the injured area; yellow arrowheads point to *mpx:*GFP^+^ cells. Scale bars, 100 μm. Source data

Genes encoding MyD88 and MyD88 pathway-related signaling components are expressed in a wide range of tissues and cell populations even in the absence of tissue damage and infection^15,30,31^. To begin to understand how MyD88 signaling affects different cell populations during cardiac regeneration in zebrafish, we dissociated cryoinjured ventricles from *myd88* LOF mutants (*myd88*^−/−^)^32^ and wild-type (WT) (*myd88*^+/+^) siblings, sorted live cells and performed single-cell RNA sequencing (scRNA-seq) analysis (Fig. 1a and Supplementary Fig. 1a). We focused our analysis at the early time point of 24 hours post cryoinjury (hpci), when *myd88* expression peaks in the cryoinjured zebrafish heart (Supplementary Fig. 1b)^10^. In addition, as the receptors of the MyD88 signaling pathway can rapidly recognize cardiac tissue damage through binding their ligands, 24 hpci is a good time point because it is within the early acute inflammatory phase^33^. We performed quality control analysis, applied filtering cutoffs and obtained a dataset consisting of 11,735 cells (8,181 and 3,554 *myd88*^+/+^ and *myd88*^−/−^ cells, respectively). To assess cell type diversity, we took into consideration known marker genes and identified five major cell cluster populations, including endocardial cells, coronary endothelial cells (cECs), myeloid cells, epicardial and epicardium-derived cells (EPDCs) and mesenchymal cells (Fig. 1b,c)^34–36^. We did not detect a CM population, presumably because their large size was incompatible with our sample preparation pipeline as reported previously^36^. As myeloid cells activate the MyD88 signaling pathway^10,22,37,38^, we decided to perform unbiased subclustering of the myeloid population to investigate their diversity (Fig. 1d). This analysis revealed five distinct myeloid clusters, of which four expressed macrophage marker genes (for example, *mpeg1.1*, *c1qa*) at higher levels and one expressed neutrophil marker genes (for example, *mpx*, *lyz*) at higher levels (Fig. 1d and Extended Data Fig. 1a,b). We identified additional genes highly expressed in this presumed neutrophil cluster and indeed observed their enrichment in neutrophils in published datasets (Extended Data Fig. 1c)^34–36^. Comparison of the myeloid populations between *myd88*^+/+^ and *myd88*^−/−^ ventricles revealed pronounced differences, such as a strong reduction of the neutrophil and the *marco* macrophage clusters in *myd88*^−/−^ ventricles (Fig. 1d). We also observed that the expression of inflammatory genes was increased in these two clusters, suggesting that they contain pro-inflammatory cells (Fig. 1e). *myd88*^−/−^ ventricles also exhibited expansion of a macrophage subpopulation that is transcriptionally unique (enriched genes: *gpx1a*, *mrc1b*, *timp4.2*, *lxn*, *lta4h*, *csf3r*, *cfh*) and is not present in *myd88*^+/+^ ventricles (Extended Data Fig. 1d,e).

Neutrophils are known pro-inflammatory mediators and immediate responders to tissue damage^39–41^. They can shape the regenerative outcome because they influence important regeneration events, including clearance of dead cells and debris from the injured area, macrophage polarization, effective revascularization, epicardial activation and myocardial regeneration^42–47^. The MyD88 signaling axis, once activated, facilitates neutrophil recruitment^22,23,37,48^. To further investigate the neutrophil phenotype observed in cryoinjured *myd88*^−/−^ ventricles (Fig. 1d), we used the *TgBAC(mpx:GFP)* neutrophil reporter line to determine neutrophil numbers after injury. While at 6 hpci we did not observe obvious differences between *myd88*^−/−^ and *myd88*^+/+^ sibling ventricles (Extended Data Fig. 1f,g), at 24 hpci neutrophil numbers were significantly reduced in *myd88*^−/−^ ventricles and their numbers remained reduced at least until 96 hpci (Fig. 1f,g). We also counted the number of neutrophils at 6 days post sham (dps) surgery and 7 days post cryoinjury (dpci), but did not observe any significant differences at these time points (Extended Data Fig. 1f,g). Together these data indicate that neutrophil numbers were reduced at 24 and 96 hpci in *myd88*^−/−^ ventricles.

As with neutrophils, the MyD88 signaling axis contributes to macrophage recruitment^10,37,38,49^. Thus, we used the *Tg(mpeg1:EGFP)* macrophage reporter line to determine whether macrophage numbers were also affected in cryoinjured *myd88*^−/−^ ventricles. Both scRNA-seq analysis and immunostaining for *mpeg1:*EGFP^+^ cells revealed that total macrophage numbers were not affected in *myd88*^−/−^ ventricles at 24 hpci (Fig. 1h,i and Extended Data Fig. 1h). While we did not observe a significant reduction in total macrophage numbers at 24 hpci, our transcriptomic data showed that some macrophage populations might be affected, with the *marco* macrophage population being severely reduced and the *hp-1* macrophage population clearly increased in *myd88*^−/−^ ventricles (Fig. 1d and Extended Data Fig. 1d,e). As the macrophage response initiates and develops later than the neutrophil response after injury, we set out to analyze cryoinjured ventricles at a later time point, when a macrophage phenotype might be more obvious. To this end, we selected 96 hpci, a time point when macrophage numbers increases significantly in WT zebrafish^10,34,50,51^, and indeed found a reduction in *mpeg1:*EGFP^+^ cells in *myd88*^−/−^ ventricles compared with *myd88*^+/+^ siblings (Fig. 1h,i).

Taken together, these transcriptomic analyses and immunostaining data reveal that during zebrafish cardiac regeneration, MyD88 is important for neutrophil and macrophage recruitment and for enhancing the inflammatory state of immune cells.

### MyD88 signaling attenuates fibrosis in injured ventricles

**Fig. 2:**
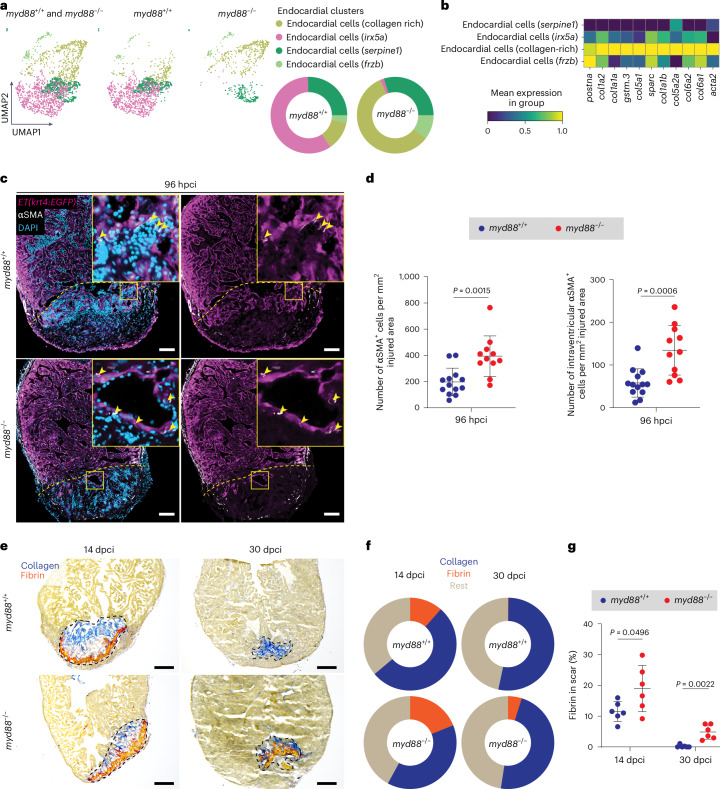
Fibrotic phenotype in cryoinjured *myd88*^−/−^ ventricles. **a**, UMAP representation of the endocardial subclusters from the scRNA-seq analysis. The pie charts show the proportions of different endocardial subclusters. **b**, Heatmap showing fibrotic gene (*postna*, *col1a2*, *col1a1a*, *gstm.3*, *col5a1*, *sparc*, *col1a1b*, *col5a2a*, *col6a2*, *col6a1*, *acta2*) expression levels in the endocardial subclusters. **c**, Representative images of immunostaining for EGFP (endocardial cells, magenta) and αSMA (myofibroblasts, white) with DAPI (DNA marker, blue) counterstaining on sections of cryoinjured *ET(krt4:EGFP); myd88*^+/+^ and *ET(krt4:EGFP); myd88*^−/−^ ventricles at 96 hpci. **d**, Total number of αSMA^+^ cells and intraventricular αSMA^+^ cells in *myd88*^+/+^ and *myd88*^−/−^ injured tissues at 96 hpci. The dots in the graphs represent individual ventricles; data are shown as the mean ± s.d.; *n* = 13 *myd88*^+/+^ and *n* = 11 *myd88*^−/−^. Statistical tests: Student’s *t*-test. **e**, Representative images of AFOG staining on sections of cryoinjured *myd88*^+/+^ and *myd88*^−/−^ ventricles at 14 and 30 dpci. **f**, Pie charts showing the proportion of scar components (collagen, blue; fibrin, red; rest of cells and tissue, light brown) in *myd88*^+/+^ and *myd88*^−/−^ scars; *n* = 6 *myd88*^+/+^ and *n* = 6 *myd88*^−/−^ for both 14 and 30 dpci. **g**, Graph showing the percentage of the fibrin/scar area at 14 and 30 dpci. The dots in the graph represent individual ventricles; data are shown as the mean ± s.d.; *n* = 6 *myd88*^+/+^ and *n* = 6 *myd88*^−/−^. Statistical test: Student’s *t*-test. The yellow dashed lines delineate the injured area and the black dashed lines the scar area; the yellow arrowheads point to αSMA^+^ cells. Scale bars, 100 μm (**c**), 200 μm (**e**). Source data

**Extended Data Fig. 2:**
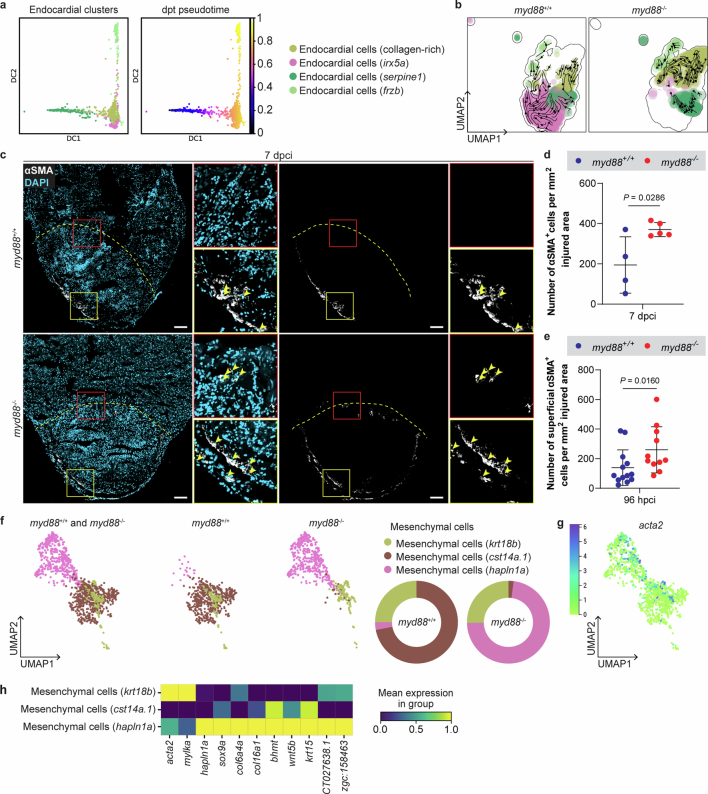
Fibrotic cell populations in cryoinjured *myd88*^*−/−*^ ventricles. **a**, Trajectory analysis revealing a potential transition from the *serpine1* endocardial cluster to the collagen-rich, *irx5a*, and *frzb* endocardial clusters. **b**, Velocity analysis done by comparing pre-mRNA and mRNA levels to infer the relations between the cells in the endocardial clusters. **c**, Representative images of immunostaining for αSMA (myofibroblasts, white) with DAPI (DNA marker, blue) counterstaining on sections of cryoinjured *myd88*^+/+^ and *myd88*^*−/−*^ ventricles at 7 dpci. **d**, Total αSMA^+^ cell numbers in *myd88*^+/+^ and *myd88*^*−/−*^ injured tissues at 7 dpci. The dots in the graph represent individual ventricles; data are shown as the mean ± s.d.; n = 4 *myd88*^+/+^ and n = 5 *myd88*^*−/−*^. Statistical test: Student’s *t*-test. **e**, Superficially localized αSMA^+^ cell numbers in *myd88*^+/+^ and *myd88*^*−/−*^ injured tissues at 96 hpci. The dots in the graph represent individual ventricles; data are shown as the mean ± s.d.; n = 13 *myd88*^+/+^ and n = 11 *myd88*^*−/−*^. Statistical test: Mann-Whitney *U*-test. **f**, UMAP representation of the mesenchymal subclusters from the scRNA-seq analysis. **g**, UMAP representation of *acta2* expression levels in the mesenchymal subclusters. **h**, Heatmap showing gene expression levels in the mesenchymal subclusters. Yellow dashed lines delineate the injured area; yellow arrowheads point to αSMA^+^ cells. Scale bars, 100 μm. Source data

Besides analyzing myeloid cells, the most extensively studied activators of the MyD88 signaling pathway^10,22,37,38^, we wanted to determine which other cell types were important for MyD88 function during cardiac regeneration. As reported previously, endothelial cells express *myd88* and components of the MyD88 signaling axis, such as the *tlr* genes^52,53^. To investigate the state and role of endocardial cells in the absence of MyD88 function during regeneration, we performed unbiased subclustering analysis of the endocardial population in our scRNA-seq dataset. This analysis revealed the presence of four distinct endocardial clusters, represented in different proportions in cryoinjured *myd88*^−/−^ and *myd88*^+/+^ ventricles (Fig. 2a). Specifically, the most abundant endocardial cell cluster found in *myd88*^+/+^ ventricles (*irx5a* endocardial cells) was significantly reduced in *myd88*^−/−^ ventricles. Interestingly, the most abundant endocardial cluster in *myd88*^−/−^ ventricles displayed high expression levels of genes encoding collagens (*col1a2*, *col1a1a*, *col5a1*, *col1a1b*, *col5a2a*, *col6a2*, *col6a1*) and genes associated with fibrosis (*postna*, *gstm.3*, *sparc*, *acta2*)^34,54^. Hence, we annotated this cluster as ‘collagen-rich endocardial cells’ (Fig. 2a,b). Pseudotime trajectory analysis indicated that cells transition from the wound endocardial cluster *serpine1* (ref. ^55^) to the other endocardial clusters (Extended Data Fig. 2a). To investigate the relationship between endocardial clusters, we also performed velocity analysis. The resulting data indicate that endocardial cells in *myd88*^−/−^ ventricles tend to progress toward the collagen-rich endocardial state (Extended Data Fig. 2b). As shown previously in mouse^56–58^ and zebrafish^35,59^, the endocardium contributes to the activated fibroblast cell population and to α-smooth muscle actin (αSMA)^+^ myofibroblasts in injured hearts; thus, increased expression of collagen and fibrotic genes in the cryoinjured *myd88*^−/−^ endocardium (Fig. 2a,b) could reflect an increase in these endocardium-derived cells. αSMA^+^ myofibroblasts are the key contributors to ECM fibrotic remodeling and scar formation in the injured heart^56,58,60^. Moreover, in the context of MyD88 signaling, MyD88 depletion in T cells results in increased cardiac fibroblast to myofibroblast transformation in vitro, as indicated by elevated αSMA and collagen type 1 expression in fibroblasts^29^. To investigate the role of MyD88 in myofibroblast differentiation during zebrafish cardiac regeneration, we immunostained for αSMA expression on sections of cryoinjured ventricles carrying the *ET(krt4:EGFP)* endocardial enhancer trap line (Fig. 2c). At 96 hpci, we found an increased abundance of αSMA^+^ cells in *myd88*^−/−^ compared with *myd88*^+/+^ siblings (Fig. 2c,d), which remained elevated until 7 dpci (Extended Data Fig. 2c,d). To differentiate between αSMA^+^ cells of different origins, we quantified intraventricularly localized αSMA^+^ cells, presumably of endocardial and fibroblast origin, and excluded superficially localized αSMA^+^ cells, presumably of epicardial, fibroblast and perivascular origin^35,61^. In line with the fibrotic transcriptomic profile of the *myd88*^−/−^ endocardium (Fig. 2a,b), at 96 hpci we found an increased number of intraventricular αSMA^+^ cells in *myd88*^−/−^ zebrafish compared with *myd88*^+/+^ siblings (Fig. 2d). Superficially localized αSMA^+^ cells were also elevated in cryoinjured *myd88*^*−/−*^ ventricles (Extended Data Fig. 2e), but not to the same extent as the intraventricularly localized αSMA^+^ cells. Altogether, these data indicate that MyD88 suppresses myofibroblast differentiation during zebrafish cardiac regeneration.

Activation of MyD88 signaling in mesenchymal stem cells reportedly conferred cardioprotective effects via Stat3 in a mouse model of cardiac ischemia–reperfusion injury^25^. As myofibroblasts, a mesenchymal cell type, are affected in *myd88*^−/−^ ventricles, we decided to take a closer look at the mesenchymal population in our scRNA-seq dataset. Unbiased subclustering analysis indicated the presence of three mesenchymal subclusters. Strikingly, strong differences between the *myd88*^−/−^ and *myd88*^+/+^ samples resulted in one cluster (*hapln1a* mesenchymal cells) being of almost completely *myd88*^−/−^ and another (*cst14a.1* mesenchymal cells) being of almost completely *myd88*^+/+^ identity (Extended Data Fig. 2f). Specifically, the *myd88*^−/−^ *hapln1a* mesenchymal cell cluster exhibited increased expression of endothelial-to-mesenchymal transition and fibrotic-related genes, such as *sox9a* and *acta2* (Extended Data Fig. 2g,h)^59^.

**Extended Data Fig. 3:**
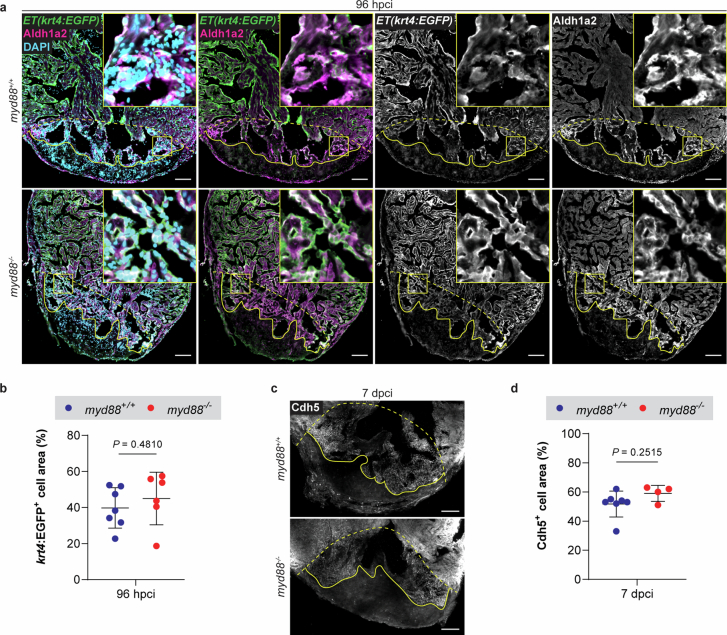
Endocardial area coverage is not affected in cryoinjured *myd88*^*−/−*^ ventricles. **a**, Representative images of immunostaining for EGFP (endocardial cells, green) and Aldh1a2 (endocardial and epicardial cell activation marker, magenta) with DAPI (DNA marker, blue) counterstaining on sections of cryoinjured *ET(krt4:EGFP); myd88*^+/+^ and *ET(krt4:EGFP); myd88*^*−/−*^ ventricles at 96 hpci. **b**, Ratio of *krt4*:EGFP^+^ cell area within the injured tissue in cryoinjured *myd88*^+/+^ and *myd88*^*−/−*^ ventricles at 96 hpci. The dots in the graph represent individual ventricles; data are shown as the mean ± s.d.; n = 7 *myd88*^+/+^ and n = 6 *myd88*^*−/−*^. Statistical test: Student’s *t*-test. **c**, Representative images of immunostaining for Cdh5 (endothelial cell marker, white) on 50-μm-thick sections of cryoinjured *myd88*^+/+^ and *myd88*^*−/−*^ ventricles at 7 dpci. **d**, Ratio of Cdh5^+^ cell area within the injured tissue in cryoinjured *myd88*^+/+^ and *myd88*^*−/−*^ ventricles at 7 dpci. The dots in the graph represent individual ventricles; data are shown as the mean ± s.d.; n = 7 *myd88*^+/+^ and n = 4 *myd88*^*−/−*^. Statistical test: Mann-Whitney *U*-test. Yellow dashed lines delineate the injured area. Scale bars, 100 μm. Source data

As our data pointed to an endocardial-related fibrotic phenotype in cryoinjured *myd88*^−/−^ ventricles, we next analyzed additional endocardial processes that could be affected in the absence of *myd88* function. Endocardial hyperinvasion of the injured area has been linked with increased endothelial-to-mesenchymal transition and fibrotic remodeling^59^. However, we did not observe any obvious differences between *myd88*^−/−^ and *myd88*^+/+^ siblings with respect to the *krt4:*EGFP^+^ and Cdh5^+^ endocardial cell area covering the injury at 96 hpci (Extended Data Fig. 3a,b) or 7 dpci (Extended Data Fig. 3c,d), respectively. Additionally, the Aldh1a2 activation pattern was similar in *myd88*^−/−^ and *myd88*^+/+^ sibling ventricles at 96 hpci (Extended Data Fig. 3a)^55^, indicating that only endothelial-to-mesenchymal transition processes were affected in cryoinjured *myd88*^−/−^ ventricles.

**Extended Data Fig. 4:**
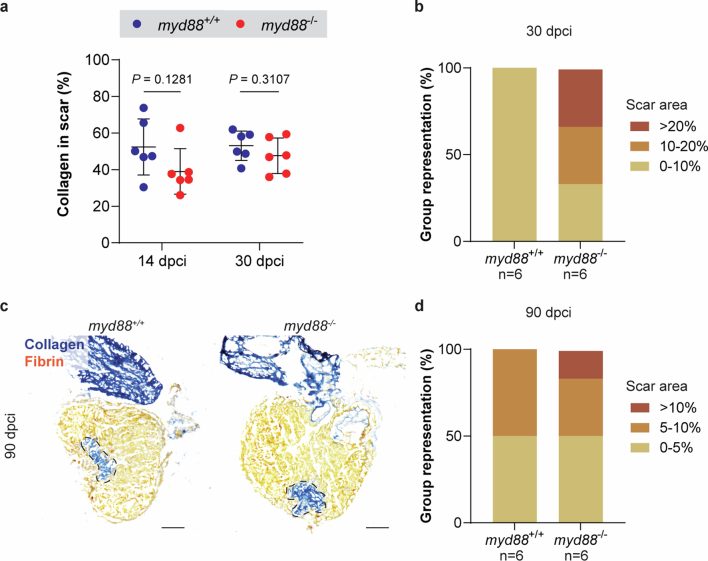
Bigger scars at 30 but not at 90 dpci in cryoinjured *myd88*^*−/−*^ ventricles. **a**, Graph showing the percentage of the collagen/scar area at 14 and 30 dpci. The dots in the graph represent individual ventricles, and data are shown as the mean ± s.d.; n = 6 *myd88*^+/+^ and n = 6 *myd88*^*−/−*^. Statistical tests: Student’s *t*-test. **b**, Graph showing representation of groups (*y* axis) of different scar area sizes (different colors) at 30 dpci in cryoinjured *myd88*^+/+^ and *myd88*^*−/−*^ ventricles. **c**, Representative images of AFOG staining on sections of cryoinjured *myd88*^+/+^ and *myd88*^*−/−*^ ventricles at 90 dpci. **d**, Graph showing representation of groups (*y* axis) of different scar area sizes (different colors) at 90 dpci in cryoinjured *myd88*^+/+^ and *myd88*^*−/−*^ ventricles. Black dashed lines delineate the scar area. Scale bars, 200 μm. Source data

Blocking MyD88 signaling in uninjured murine hearts leads to increased fibrosis^28^. To investigate the regenerative potential and scar resolution abilities of cryoinjured *myd88*^−/−^ ventricles, we performed acid fuchsin orange G (AFOG) staining. As reported previously, scar tissue in 14 dpci WT hearts consists of an extensive collagen network and a ring-like peripheral fibrin structure^5,33,62^. In contrast, the scar tissue in *myd88*^−/−^ ventricles at 14 dpci displayed thicker fibrin-rich areas in comparison with their *myd88*^+/+^ siblings (Fig. 2e), and these increased fibrin levels persisted until at least until 30 dpci (Fig. 2e–g). However, the collagen proportions within the scars remained similar between *myd88*^−/−^ and *myd88*^+/+^ siblings (Extended Data Fig. 4a). Additionally, at 30 dpci, there were more *myd88*^−/−^ ventricles with bigger scar areas compared with their *myd88*^+/+^ siblings (Extended Data Fig. 4b). We also examined scars at 90 dpci to analyze fibrotic tissue persistence, but did not observe significant differences at this time point (Extended Data Fig. 4c,d).

In summary, the fibrotic-like transcriptomic profile (enrichment in collagen and fibrotic genes) observed in both endocardial and mesenchymal cells, along with the increased number of total and intraventricular αSMA^+^ cells, increased fibrin levels and larger scars in cryoinjured *myd88*^−/−^ hearts, collectively indicate that MyD88 signaling has a role in limiting fibrosis during zebrafish cardiac regeneration.

### Endocardial MyD88 signaling activates the PI3K/AKT pathway

**Fig. 3:**
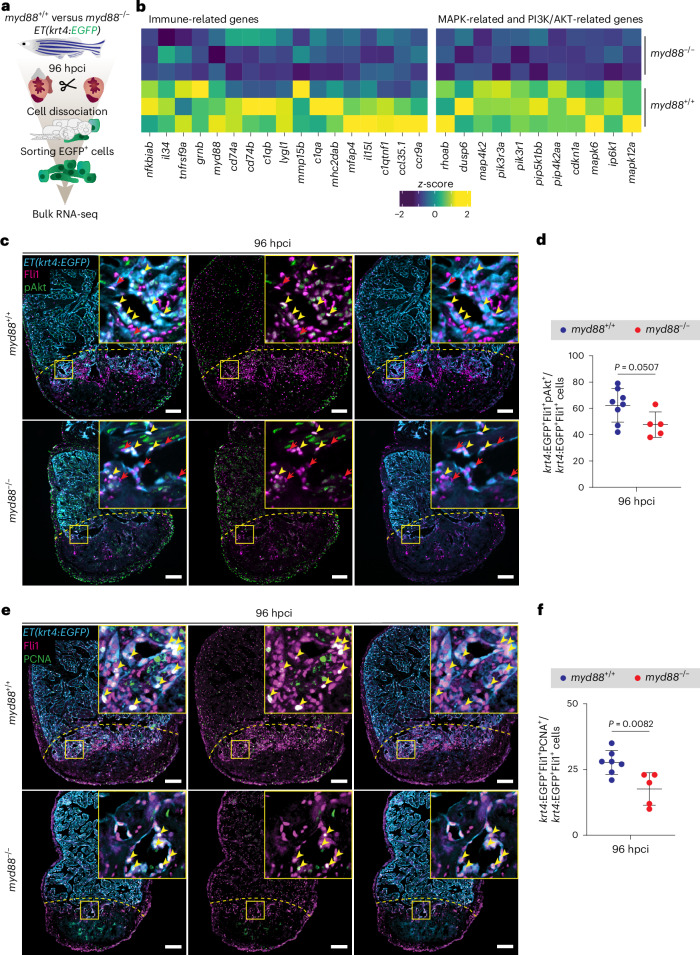
The PI3K/AKT signaling pathway is suppressed in the *myd88*^−/−^ endocardium. **a**, Experimental plan for bulk RNA-seq analysis on sorted endocardial cells from *ET(krt4:EGFP); myd88*^+/+^ and *ET(krt4:EGFP); myd88*^−/−^ ventricles at 96 hpci. **b**, Heatmap showing differential expression of significantly (*P* < 0.05) downregulated genes in the *myd88*^−/−^ endocardium at 96 hpci. **c**, Representative images of immunostaining for EGFP (endocardial cells, blue), Fli1 (endothelial cell nuclei, magenta) and pAkt (phosphoAkt, green) on sections of cryoinjured *ET(krt4:EGFP); myd88*^+/+^ and *ET(krt4:EGFP); myd88*^−/−^ ventricles at 96 hpci. **d,** *krt4*:EGFP^+^Fli1^+^pAkt^+^/*krt4*:EGFP^+^Fli1^+^ cell percentage in *myd88*^+/+^ and *myd88*^−/−^ 50-μm-wide areas on the basal-most side of the injured tissue at 96 hpci. The dots in the graph represent individual ventricles; data are shown as the mean ± s.d.; *n* = 8 *myd88*^+/+^ and *n* = 5 *myd88*^−/−^. Statistical test: Student’s *t*-test. **e**, Representative images of immunostaining for EGFP (endocardial cells, blue), Fli1 (endothelial cell nuclei, magenta) and PCNA (proliferation marker, green) on sections of cryoinjured *ET(krt4:EGFP); myd88*^+/+^ and *ET(krt4:EGFP); myd88*^−/−^ ventricles at 96 hpci. **f**, *krt4*:EGFP^+^Fli1^+^PCNA^+^/*krt4*:EGFP^+^Fli1^+^ cell percentage in *myd88*^+/+^ and *myd88*^−/−^ 50-μm-wide areas on the basal-most side of the injured tissue at 96 hpci. The dots in the graph represent individual ventricles; data are shown as the mean ± s.d.; *n* = 7 *myd88*^+/+^ and n = 5 *myd88*^−/−^. Statistical test: Student’s *t*-test. The yellow dashed lines delineate the injured area; the yellow arrowheads point to *krt4*:EGFP^+^Fli1^+^pAkt^+^ (**c**) and *krt4*:EGFP^+^Fli1^+^PCNA^+^ (**e**) cells; the red arrowheads (**c**) point to *krt4*:EGFP^+^Fli1^+^pAkt^−^ cells. Scale bars, 100 μm. Source data

**Extended Data Fig. 5:**
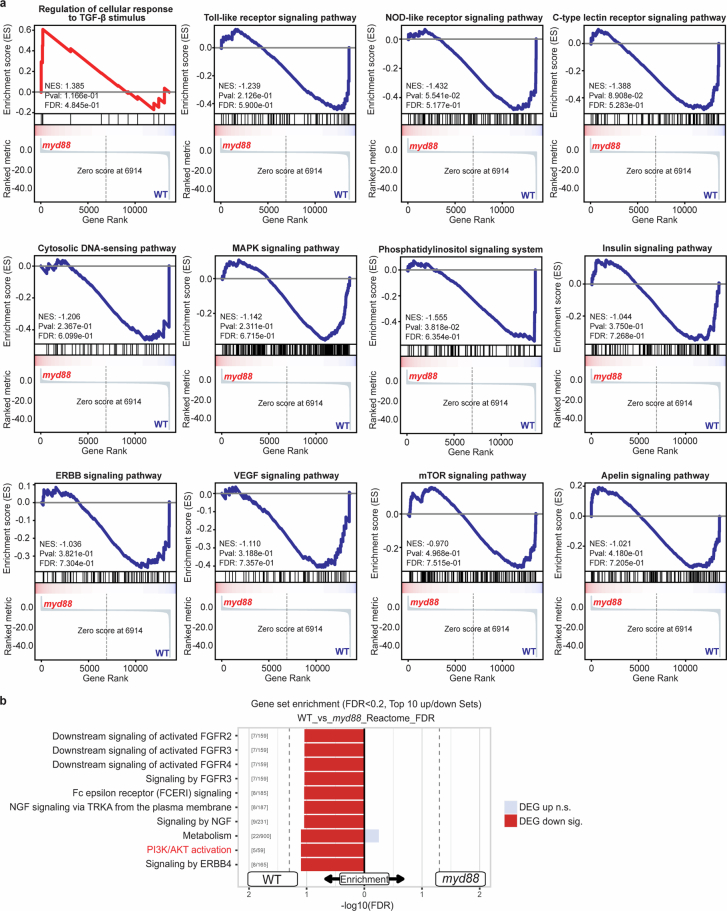
Enrichment analysis terms affected in the cryoinjured *myd88*^*−/−*^ endocardium at 96 hpci. **a**, Selected GSEA plots for GO and KEGG pathway terms from transcriptomic analysis in the cryoinjured *myd88*^*−/−*^ versus *myd88*^+/+^ endocardium at 96 hpci. Plots with red and blue lines indicate processes enriched in *myd88* (mutant; *myd88*^*−/−*^) and WT (*myd88*^+/+^), respectively. **b**, Plot showing Reactome pathway terms enriched in the cryoinjured *myd88*^+/+^ (WT) versus *myd88*^*−/−*^ (*myd88* mutant) endocardium at 96 hpci. Source data

**Extended Data Fig. 6:**
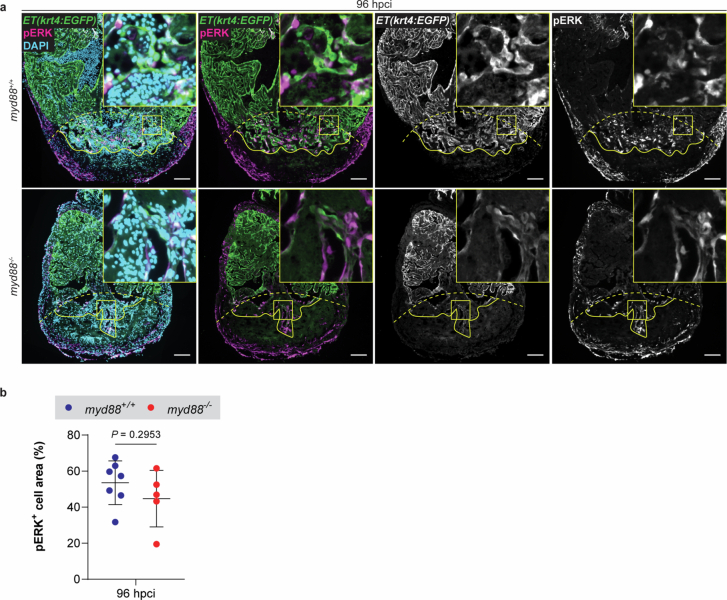
pERK^+^ area coverage is not significantly affected in cryoinjured *myd88*^*−/−*^ ventricles. **a**, Representative images of immunostaining for EGFP (endocardial cells, green) and pERK (activated MAPK signaling pathway, magenta) with DAPI (DNA marker, blue) counterstaining on sections of cryoinjured *ET(krt4:EGFP); myd88*^+/+^ and *ET(krt4:EGFP); myd88*^*−/−*^ ventricles at 96 hpci. **b**, Ratio of pERK^+^ cell area within the injured tissue in cryoinjured *myd88*^+/+^ and *myd88*^*−/−*^ ventricles at 96 hpci. The dots in the graph represent individual ventricles; data are shown as the mean ± s.d.; n = 7 *myd88*^+/+^ and n = 5 *myd88*^*−/−*^. Statistical test: Student’s *t*-test. Yellow dashed lines delineate the injured area. Scale bars, 100 μm. Source data

To gain mechanistic insights into how MyD88 signaling regulates endocardial-specific processes during regeneration, we sorted endocardial cells from *myd88*^−/−^ and *myd88*^+/+^ siblings using the *ET(krt4:EGFP)* line at 96 hpci and conducted bulk RNA-seq analysis (Fig. 3a and Supplementary Fig. 2). In line with the previously observed fibrotic phenotype (Fig. 2), gene set enrichment analysis (GSEA) revealed an increase in the regulation of the cellular response to transforming growth factor-β stimulus in the cryoinjured *myd88*^−/−^ endocardium (Extended Data Fig. 5a). Additionally, GSEA (Extended Data Fig. 5a) and closer examination of the most differentially expressed genes (Fig. 3b) revealed that immune-associated processes, such as TLR, nucleotide-binding oligomerization domain (NOD)-like receptor, C-type lectin receptor and cytosolic DNA-sensing signaling pathways, as well as the mitogen-activated protein kinase (MAPK) signaling pathway, were impaired in the *myd88*^−/−^ endocardium. As the MAPK signaling pathway has been linked with the activated endocardium in both the cardiac resection^63,64^ and cryoinjury^51^ models, we immunostained for phosphoERK (pERK), an indicator of activated MAPK signaling, in *ET(krt4:EGFP)* ventricles and verified its presence in the endocardium at 96 hpci. However, we did not observe statistically significant differences in the pERK^+^ cell area covering the injury between *myd88*^−/−^ and *myd88*^+/+^ siblings (Extended Data Fig. 6a,b), indicating that defective MAPK signaling was not responsible for the endocardial phenotype observed in *myd88*^−/−^ ventricles. This observation is in line with a previous study where lipopolysaccharide treated *Myd88*^−/−^ mice displayed activation of the MAPK signaling pathway^65^.

GSEA and examination of the most differentially expressed genes (DEGs) (Fig. 3b, Supplementary Table 1 and Extended Data Fig. 5a,b) further indicated that activation of the PI3K/AKT pathway is impaired in the *myd88*^−/−^ endocardium. Additionally, GSEA terms related to upstream (ErBB, IGF, VEGF, FGF and NGF pathways) and downstream components (mTOR pathway) of PI3K/AKT signaling were substantially downregulated in the cryoinjured *myd88*^−/−^ endocardium (Extended Data Fig. 5a,b)^64,66–69^. Interestingly, MyD88 signaling can also regulate PI3K/AKT pathway activation^28,70,71^. Hence, we costained for phospho-Akt (pAkt), which indicates activation of PI3K/AKT signaling when present in the nucleus, together with the nuclear endothelial cell marker Fli1 in sections of *ET(krt4:EGFP)* ventricles and found a tendency for reduced activation of the PI3K/AKT pathway in *myd88*^−/−^ endocardial cells at 96 hpci (Fig. 3c,d). Given that PI3K/AKT activation affects cell proliferation^68,72^, we then assessed endocardial proliferation at 96 hpci, when it reaches high levels in WT^55,68^. Notably, we found significantly reduced proliferation in *myd88*^−/−^ endocardial cells at 96 hpci (Fig. 3e,f).

In summary, these findings underscore the critical role of MyD88 in regulating signaling cascades in the injured endocardium. Particularly, we identified impairments in the immune response, in the activation of the PI3K/AKT pathway and in endocardial cell proliferation in cryoinjured *myd88*^−/−^ ventricles.

### MyD88 signaling promotes revascularization and CM repopulation

**Extended Data Fig. 7:**
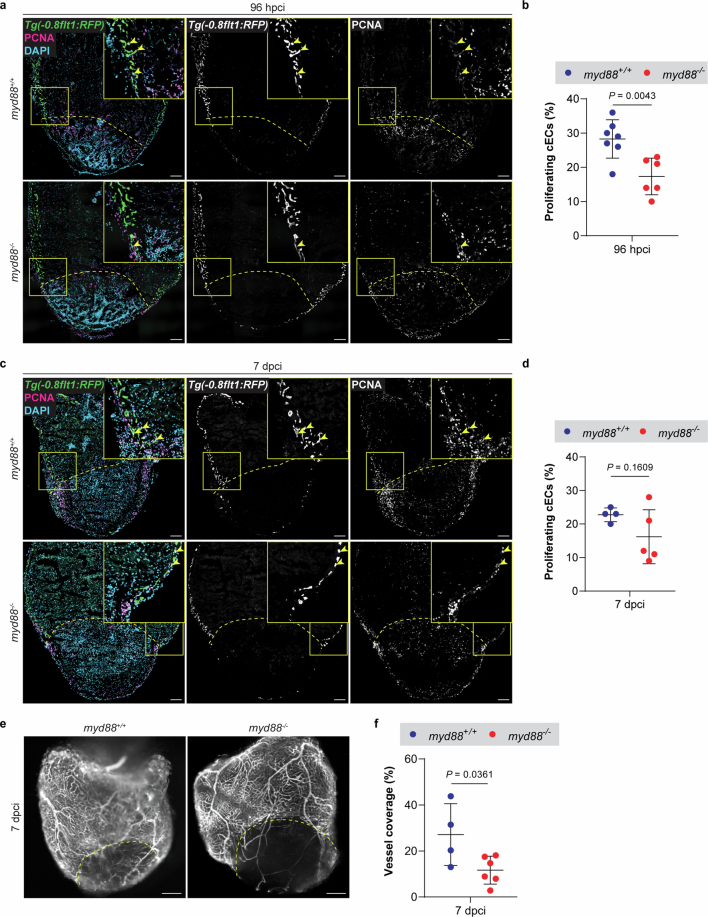
Revascularization phenotype in cryoinjured *myd88*^*−/−*^ ventricles. **a, c**, Representative images of immunostaining for RFP (coronary endothelial cells, green) and PCNA (proliferation marker, magenta) with DAPI (DNA marker, blue) counterstaining on sections of cryoinjured *Tg(-0.8flt1:RFP); myd88*^+/+^ and *Tg(-0.8flt1:RFP); myd88*^*−/−*^ ventricles at 96 hpci (a) and 7 dpci (c). **b, d**, Percentage of proliferating coronary endothelial cells in the injured tissues and border zone areas (200 μm) at 96 hpci (b) and 7 dpci (d). The dots in the graphs represent individual ventricles; data are shown as the mean ± s.d.; n = 7 *myd88*^+/+^, n = 6 *myd88*^*−/−*^ at 96 hpci (b), and n = 4 *myd88*^+/+^, n = 5 *myd88*^*−/−*^ at 7 dpci (d). Statistical tests: Student’s *t*-test. **e**, Wholemount images of cryoinjured *Tg(-0.8flt1:RFP); myd88*^+/+^ and *Tg(-0.8flt1:RFP); myd88*^*−/−*^ ventricles at 7 dpci. **f**, Percentage of coronary vessel coverage in the injured tissues of cryoinjured *Tg(-0.8flt1:RFP); myd88*^+/+^ and *Tg(-0.8flt1:RFP); myd88*^*−/−*^ ventricles at 7 dpci. The dots in the graphs represent individual ventricles; data are shown as the mean ± s.d.; n = 4 *myd88*^+/+^ and n = 6 *myd88*^*−/−*^. Statistical test: Student’s *t*-test. Yellow dashed lines delineate the injured area; yellow arrowheads point to proliferating coronary endothelial cells. Scale bars, 100 μm (a,c), 200 μm (e). Source data

Revascularization is one of the earliest processes observed in the injured area and it is vital for effective cardiac tissue regeneration^73,74^. Following cryoinjury of the zebrafish ventricle, cECs undergo proliferation and initiate the sprouting of new vessels, both superficially and intraventricularly. While intraventricular revascularization is orchestrated by endocardial vascular endothelial growth factor A signaling, superficial revascularization is partially regulated by Apelin signaling^74^. We found that both endocardial VEGF and Apelin signaling pathways were impaired in cryoinjured *myd88*^−/−^ ventricles (Extended Data Fig. 5a). Additionally, neutrophils, which were significantly reduced in *myd88*^−/−^ ventricles (Fig. 1d,f,g), express VEGF and promote revascularization^42,44,46^. Hence, we used the *Tg(-0.8flt1:RFP)* reporter line, which labels cECs and assessed their proliferation at 96 hpci, when cEC proliferation peaks in cryoinjured WT ventricles^74^. We found that cEC proliferation was significantly reduced at 96 hpci in *myd88*^−/−^ hearts when compared with *myd88*^+/+^ siblings (Extended Data Fig. 7a,b). While cEC proliferation recovered by 7 dpci (Extended Data Fig. 7c,d), coronary vessel coverage in the injured area remained impaired at 7 dpci (Extended Data Fig. 7e,f) in *myd88*^−/−^. Together, these data reveal that revascularization of the injured area was affected in cryoinjured *myd88*^−/−^ ventricles.

**Fig. 4:**
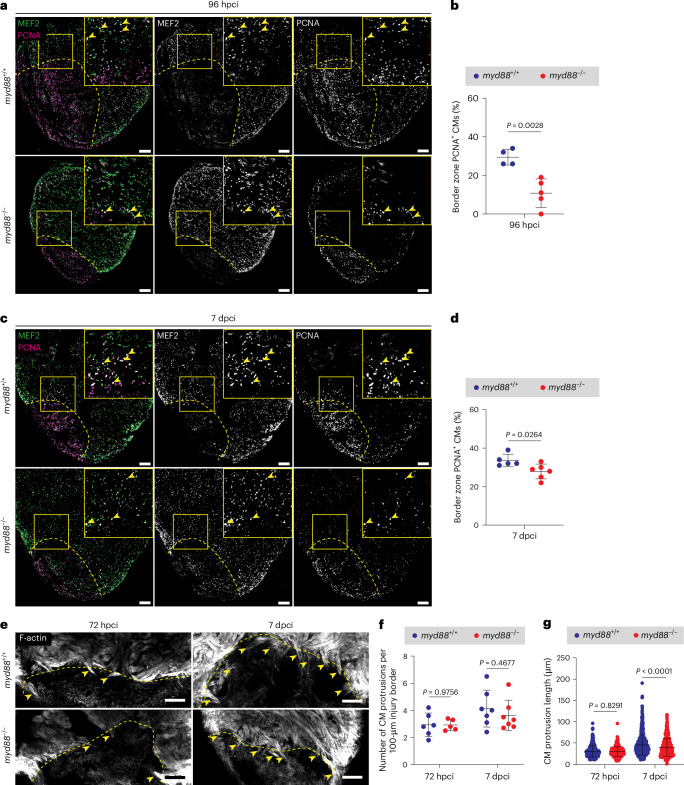
Reduced CM proliferation and reduced length of CM protrusions toward the injured tissue in cryoinjured *myd88*^−/−^ ventricles. **a**,**c**, Representative images of immunostaining for MEF2 (CM nuclei, green) and PCNA (proliferation marker, magenta) on sections of cryoinjured *myd88*^+/+^ and *myd88*^−/−^ ventricles at 96 hpci (**a**) and 7 dpci (**c**). **b**,**d**, Quantification of proliferating CMs in border zone areas (100 μm) at 96 hpci (**b**) and 7 dpci (**d**). The dots in the graphs represent individual ventricles; data are shown as the mean ± s.d.; *n* = 4 *myd88*^+/+^ and *n* = 5 *myd88*^−/−^ (**b**); *n* = 5 *myd88*^+/+^ and *n* = 6 *myd88*^−/−^ (**d**). Statistical tests: Student’s *t*-test. **e**, Representative images of phalloidin staining for F-actin (white) on 50-μm-thick sections of cryoinjured *myd88*^+/+^ and *myd88*^−/−^ ventricles at 72 hpci and 7 dpci. **f**, Quantification of the number of CM protrusions. The dots in the graph represent individual ventricles; data are shown as the mean ± s.d.; *n* = 6 *myd88*^+/+^ and *n* = 5 *myd88*^−/−^ for 72 hpci; *n* = 7 *myd88*^+/+^ and *n* = 7 *myd88*^−/−^ for 7 dpci. Statistical tests: Student’s *t-*test. **g**, Quantification of CM protrusion length. The dots in the graph represent individual CM protrusions; data are shown as the mean ± s.d.; *n* = 366 *myd88*^+/+^ and *n* = 274 *myd88*^−/−^ for 72 hpci; *n* = 633 *myd88*^+/+^ and *n* = 459 *myd88*^−/−^ for 7 dpci. Statistical tests: Mann–Whitney *U*-test. The yellow dashed lines delineate the injured area; the yellow arrowheads point to proliferating (**a**,**c**) and protruding (**e**) CMs. Scale bars, 100 μm. Source data

**Extended Data Fig. 8:**
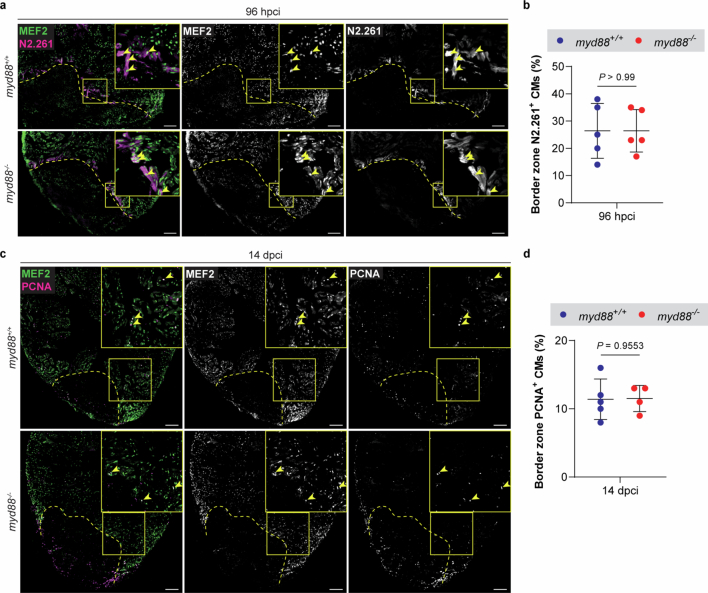
CM dedifferentiation at 96 hpci and proliferation at 14 dpci are not significantly affected in cryoinjured *myd88*^*−/−*^ ventricles. **a**, Representative images of immunostaining for MEF2 (CM nuclei, green) and N2.261 (dedifferentiation marker, magenta) on sections of cryoinjured *myd88*^+/+^ and *myd88*^*−/−*^ ventricles at 96 hpci. **b**, Quantification of the number of dedifferentiating CMs in border zone areas (100 μm) at 96 hpci. The dots in the graphs represent individual ventricles; data are shown as the mean ± s.d.; n = 5 *myd88*^+/+^ and n = 5 *myd88*^*−/−*^. Statistical test: Student’s *t*-test. **c**, Representative images of immunostaining for MEF2 (CM nuclei, green) and PCNA (proliferation marker, magenta) on sections of cryoinjured *myd88*^+/+^ and *myd88*^*−/−*^ ventricles at 14 dpci. **d**, Quantification of proliferating CMs in border zone areas (100 μm) at 14 dpci. The dots in the graphs represent individual ventricles; data are shown as the mean ± s.d.; n = 5 *myd88*^+/+^ and n = 4 *myd88*^*−/−*^. Statistical test: Student’s *t*-test. Yellow dashed lines delineate the injured area; yellow arrowheads point to dedifferentiating (a) and proliferating (c) CMs. Scale bars, 100 μm. Source data

During cardiac regeneration in zebrafish, CMs undergo dedifferentiation and proliferation to repopulate the injured tissue^75–77^ and this process is heavily influenced by the microenvironment surrounding the CMs^78^. Given the altered inflammatory and fibrotic environment in cryoinjured *myd88*^−/−^ ventricles, along with the impaired numbers and functions of various cell types that precede CM appearance in the injured area, we decided to assess CM behavior. We observed a significant reduction in CM proliferation in the injury border zone at 96 hpci and 7 dpci in *myd88*^−/−^ cryoinjured ventricles (Fig. 4a–d). We also examined CM proliferation at 14 dpci and dedifferentiation at 96 hpci^79^ but did not find any significant differences (Extended Data Fig. 8a–d). Furthermore, we quantified CM protrusive activity toward the injury at 72 hpci and 7 dpci and found that while the number of protrusions was the same between *myd88*^−/−^ and *myd88*^+/+^ siblings, CM protrusions were shorter in *myd88*^−/−^ ventricles at 7 dpci (Fig. 4e–g). These results highlight the essential role of MyD88 in facilitating efficient CM proliferation and protrusion toward the injured tissue during cardiac regeneration.

### Endothelial *myd88* overexpression reverses *myd88*^−/−^ phenotypes

**Fig. 5:**
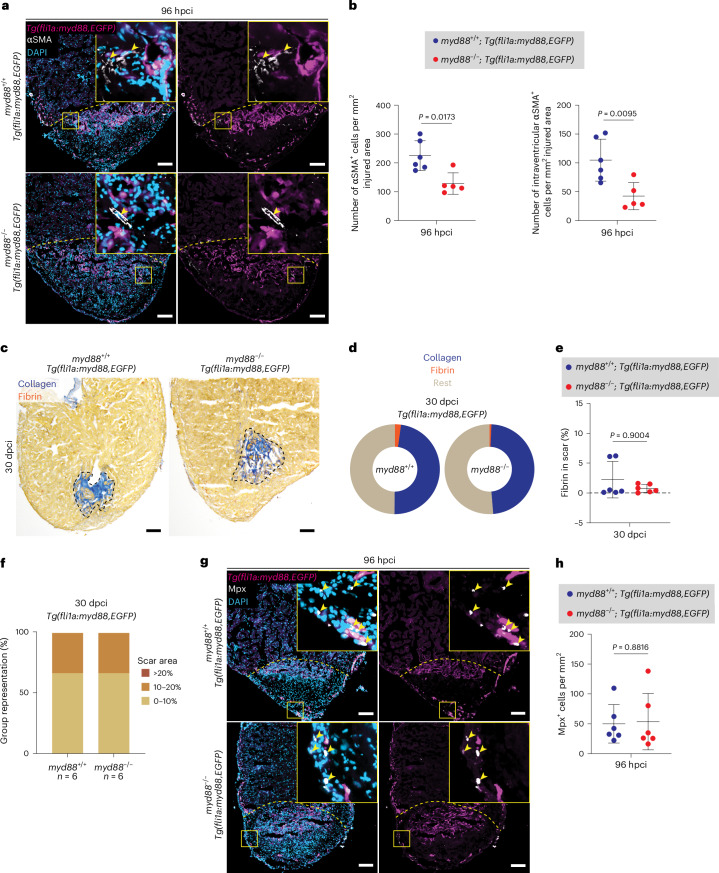
*myd88* overexpression in endothelial cells rescues the fibrotic, scarring and neutrophil phenotypes in cryoinjured *myd88*^−/−^ ventricles. **a**, Representative images of immunostaining for EGFP (endothelial cells, magenta) and αSMA (myofibroblasts, white) with DAPI (DNA marker, blue) counterstaining on sections of cryoinjured *Tg(fli1a:myd88,EGFP*); *myd88*^+/+^ and *Tg(fli1a:myd88,EGFP*); *myd88*^−/−^ ventricles at 96 hpci. **b**, Total number of αSMA^+^ cells and of intraventricular αSMA^+^ cells in *Tg(fli1a:myd88,EGFP); myd88*^*+/+*^ and *Tg(fli1a:myd88,EGFP); myd88*^−/−^ injured tissues at 96 hpci. The dots in the graphs represent individual ventricles; data are shown as the mean ± s.d.; *n* = 6 *Tg(fli1a:myd88,EGFP); myd88*^*+/+*^ and *n* = 5 *Tg(fli1a:myd88,EGFP); myd88*^−/−^. Statistical tests: Student’s *t*-test. **c**, Representative images of AFOG staining on sections of cryoinjured *Tg(fli1a:myd88,EGFP*); *myd88*^+/+^ and *Tg(fli1a:myd88,EGFP*); *myd88*^−/−^ ventricles at 30 dpci. **d**, Pie charts showing the proportion of scar components (collagen, blue; fibrin, red; rest of cells and tissue, light brown) in *Tg(fli1a:myd88,EGFP*); *myd88*^+/+^ and *Tg(fli1a:myd88,EGFP*); *myd88*^−/−^ scars; *n* = 6 *Tg(fli1a:myd88,EGFP); myd88*^*+/+*^ and *n* = 6 *Tg(fli1a:myd88,EGFP); myd88*^−/−^. **e**, Graph showing the percentage of fibrin/scar area at 30 dpci. The dots in the graph represent individual ventricles; data are shown as the mean ± s.d.; *n* = 6 *Tg(fli1a:myd88,EGFP); myd88*^*+/+*^ and *n* = 6 *Tg(fli1a:myd88,EGFP); myd88*^−/−^. Statistical test: Mann–Whitney *U*-test. **f**, Graph showing the representation of groups (*y* axis) of different scar area sizes (different colors) at 30 dpci for cryoinjured *Tg(fli1a:myd88,EGFP*); *myd88*^+/+^ and *Tg(fli1a:myd88,EGFP*); *myd88*^−/−^ ventricles. **g**, Representative images of immunostaining for EGFP (endothelial cells, magenta) and Mpx (neutrophils, white) with DAPI (DNA marker, blue) counterstaining on sections of cryoinjured *Tg(fli1a:myd88,EGFP*); *myd88*^+/+^ and *Tg(fli1a:myd88,EGFP*); *myd88*^−/−^ ventricles at 96 hpci. **h**, Mpx^+^ cell numbers in *Tg(fli1a:myd88,EGFP*); *myd88*^+/+^ and *Tg(fli1a:myd88,EGFP*); *myd88*^−/−^ injured tissues and border zone areas (100 μm) at 96 hpci. The dots in the graph represent individual ventricles; data are shown as the mean ± s.d.; *n* = 6 *Tg(fli1a:myd88,EGFP); myd88*^*+/+*^ and *n* = 6 *Tg(fli1a:myd88,EGFP); myd88*^−/−^. Statistical test: Student’s *t*-test. The yellow dashed lines delineate the injured area; the black dashed lines delineate the scar area; the yellow arrowheads point to αSMA^+^ (**a**) and Mpx^+^ (**g**) cells. Scale bars, 100 μm (**a**,**g**), 200 μm (**c**). Source data

**Extended Data Fig. 9:**
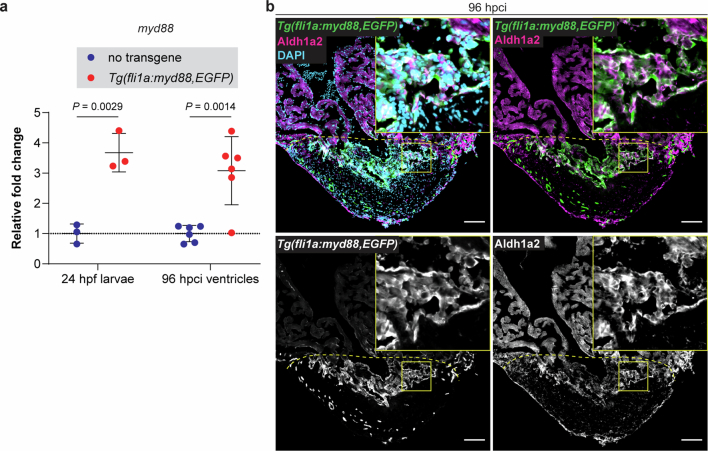
Validation of the *Tg(fli1a:myd88,EGFP)* line. **a**, RT-qPCR analysis of *myd88* mRNA levels in transgenic (*Tg(fli1a:myd88,EGFP*)) and non-transgenic 24 hpf larvae and 96 hpci ventricles. Data are shown as the mean ± s.d.; n = 3 (no transgene) and n = 3 (*Tg(fli1a:myd88,EGFP)*) 24 hpf larvae and n = 6 (no transgene) and n = 6 (*Tg(fli1a:myd88,EGFP)*) 96 hpci ventricles. Statistical tests: Student’s *t*-test. Ct values listed in Supplementary Table 3. **b**, Representative images of immunostaining for EGFP (endothelial cells, green) and Aldh1a2 (endocardial and epicardial cell activation marker, magenta) with DAPI (DNA marker, blue) counterstaining on a section of a cryoinjured *Tg(fli1a:myd88,EGFP*); *myd88*^+/+^ ventricle at 96 hpci. Yellow dashed lines delineate the injured area. Scale bars, 100 μm. Source data

To investigate whether the loss of MyD88 in endothelial cells was the contributing factor for the fibrotic phenotype observed in cryoinjured *myd88*^−/−^ ventricles, we specifically overexpressed (OE) *myd88* in endothelial cells. For this purpose, we generated a *Tg(fli1a:myd88,EGFP)* line that expresses *myd88* and *EGFP* under the control of a bidirectional *fli1a* promoter. As expected, *myd88* expression was increased in transgenic zebrafish when compared with non-transgenic sibling larvae and adult cryoinjured ventricles (Extended Data Fig. 9a), thereby validating the overexpression ability of the tool. In addition, the transgenic EGFP signal colocalized with the endocardial Aldh1a2 signal in cryoinjured ventricles, validating the endothelial-specific expression profile of the *Tg(fli1a:myd88,EGFP)* line (Extended Data Fig. 9b). We analyzed *myd88*^−/−^ and *myd88*^+/+^ siblings carrying the overexpression transgene and found that the previously elevated αSMA^+^ cells (total and intraventricular) (Fig. 2c,d) not only reverted to control levels, but also significantly decreased in cryoinjured *myd88*^−/−^ ventricles at 96 hpci (Fig. 5a,b). To assess whether endothelial-specific *myd88* overexpression could improve the regeneration potential of cryoinjured *myd88*^−/−^ ventricles, we analyzed scars at 30 dpci. Remarkably, *Tg(fli1a:myd88,EGFP)*; *myd88*^−/−^ and *myd88*^+/+^ sibling ventricles were indistinguishable in terms of fibrin abundance and scar area size (Fig. 5c–f). Altogether, these data further indicate that endothelial MyD88 function is critical to limit fibrosis in the regenerating zebrafish heart.

Endothelial cells activate immune-regulatory programs, secrete cytokines and chemokines, and facilitate early immune cell recruitment in response to cardiac injury^14,52,55,80^. They also express MyD88 and components of the MyD88 signaling axis, including TLRs^52,53^. To determine whether *myd88* overexpression in endothelial cells could also restore the low neutrophil levels observed in cryoinjured *myd88*^−/−^ ventricles (Fig. 1d,f,g), we quantified the number of Mpx^+^ cells and found that neutrophil levels were indeed restored at 96 hpci (Fig. 5g,h), indicating that MyD88 function in endothelial cells is sufficient to rescue the neutrophil phenotype. Overall, these endothelial-specific *myd88* overexpression experiments support the model that endothelial-specific MyD88 activation promotes neutrophil recruitment in the cryoinjured heart.

### MyD88-Cxcl18b signaling promotes neutrophil recruitment after cardiac injury

**Fig. 6:**
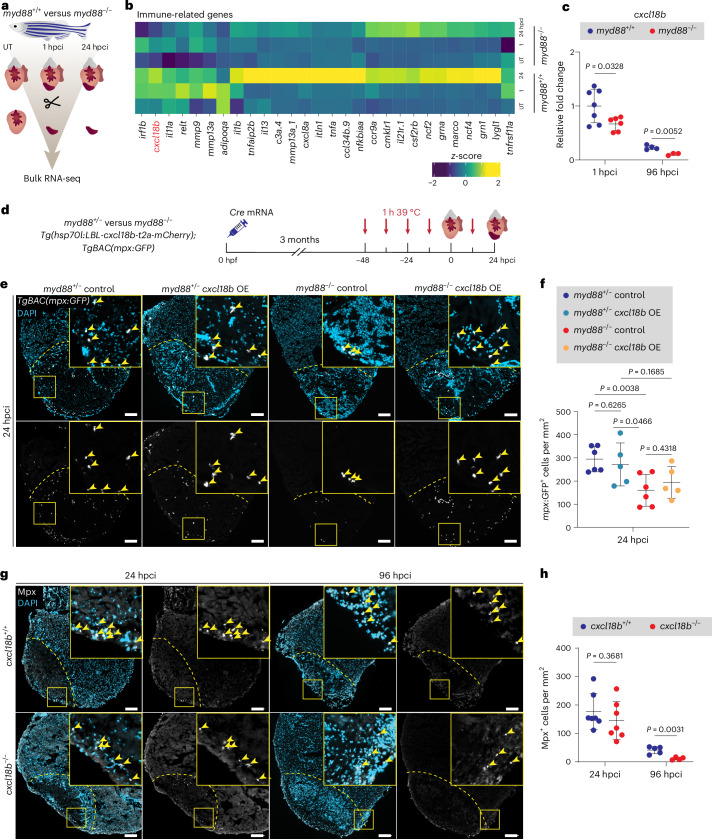
*cxcl18b* is activated by MyD88 signaling and controls neutrophil recruitment. **a**, Experimental plan for bulk RNA-seq analysis on *myd88*^+/+^ and *myd88*^−/−^ UT ventricles and injured tissues at 1 and 24 hpci. **b**, Heatmap showing differential expression of downregulated immune-related genes in *myd88*^−/−^ UT ventricles and injured tissues at 1 and 24 hpci. **c**, RT–qPCR analysis of *cxcl18b* mRNA levels in cryoinjured *myd88*^+/+^ and *myd88*^−/−^ ventricles at 1 and 96 hpci. Data are shown as the mean ± s.d.; *n* = 7 *myd88*^+/+^ and *n* = 6 *myd88*^−/−^ for 1 hpci; *n* = 4 *myd88*^+/+^ and *n* = 3 *myd88*^−/−^ for 96 hpci. Statistical tests: Student’s *t*-test. Ct values are listed in Supplementary Table 3. **d**, Experimental plan for *Cre* mRNA-injected (*cxcl18b* overexpression (OE)) or uninjected (control) *myd88*^+/−^ and *Cre* mRNA-injected (*cxcl18b* OE) or uninjected (control) *myd88*^−/−^ *Tg(hsp70l:LBL-cxcl18b-t2a-mCherry*)*; TgBAC(mpx:GFP)* siblings at 24 hpci. **e**, Representative images of immunostaining for GFP (neutrophils, white) with DAPI (DNA marker, blue) counterstaining on sections of cryoinjured *myd88*^+/−^ control, *myd88*^+/−^ *cxcl18b* OE, *myd88*^−/−^ control and *myd88*^−/−^ *cxcl18b* OE *TgBAC(mpx:GFP)* ventricles at 24 hpci. **f,** *mpx:*GFP^+^ cell numbers in injured tissues and border zone areas (100 μm) at 24 hpci. The dots in the graph represent individual ventricles; data are shown as the mean ± s.d.; *n* = 6 *myd88*^+/−^ control, *n* = 5 *myd88*^+/−^ *cxcl18b* OE, *n* = 6 *myd88*^−/−^ control and *n* = 5 *myd88*^−/−^ *cxcl18b* OE. Statistical tests: Student’s *t*-test. **g**, Representative images of immunostaining for Mpx (neutrophils, white) with DAPI (DNA marker, blue) counterstaining on sections of cryoinjured *cxcl18b*^+/+^ and *cxcl18b*^−/−^ ventricles at 24 and 96 hpci. **h**, Mpx^+^ cell numbers in *cxcl18b*^+/+^ and *cxcl18b*^−/−^ injured tissues and border zone areas (100 μm) at 24 and 96 hpci. The dots in the graphs represent individual ventricles; data are shown as the mean ± s.d.; *n* = 7 *cxcl18b*^+/+^ and *n* = 7 *cxcl18b*^−/−^ for 24 hpci; *n* = 5 *cxcl18b*^+/+^ and *n* = 4 *cxcl18b*^−/−^ for 96 hpci. Statistical tests: Student’s *t*-test. The yellow dashed lines delineate the injured area; the yellow arrowheads point to *mpx:*GFP^+^ (**e**) and Mpx^+^ (**g**) cells. Scale bars, 100 μm. Source data

**Extended Data Fig. 10:**
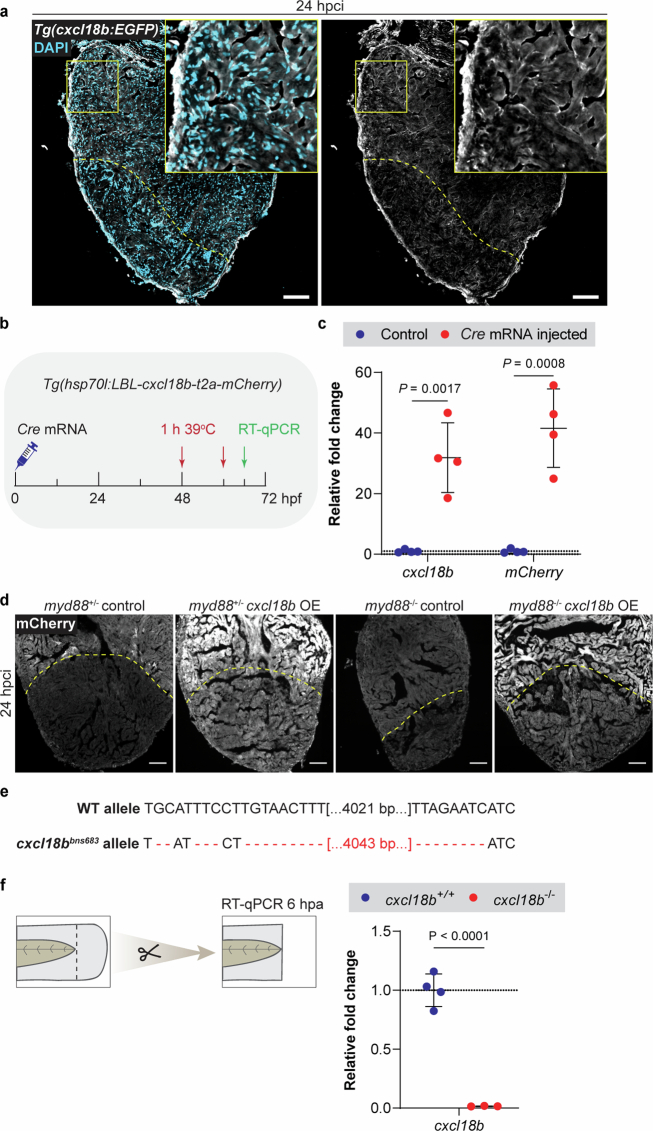
Validation of *cxcl18b* tools. **a**, Representative images of immunostaining for EGFP (*cxcl18b* expression, white) with DAPI (DNA marker, blue) counterstaining on a section of a cryoinjured *Tg(cxcl18b:EGFP)* ventricle at 24 hpci; n = 1. **b**, Experimental plan for *Cre* mRNA injected and uninjected (control) *Tg(hsp70l:LBL-cxcl18b-t2a-mCherry*) sibling larvae. **c**, RT-qPCR analysis of *cxcl18b* and *mCherry* mRNA levels in *Cre* mRNA-injected and uninjected (control) *Tg(hsp70l:LBL-cxcl18b-t2a-mCherry*) larvae. The dots in the graph represent individual larvae; data are shown as the mean ± s.d.; n = 4 (Control) and n = 4 (*Cre* mRNA injected). Statistical tests: Student’s *t*-test. Ct values listed in Supplementary Table 3. **d**, Representative images of immunostaining for mCherry (*cxcl18b* overexpressing cells, white) on sections of cryoinjured ventricles from uninjected (control) *myd88*^+/−^, *Cre* mRNA injected (*cxcl18b* OE) *myd88*^+/−^, uninjected (control) *myd88*^*−/−*^ and Cre mRNA injected (*cxcl18b* OE) *myd88*^*−/−*^ siblings at 24 hpci; all zebrafish carry the *hsp70l:LBL-cxcl18b-t2a-mCherry* transgene; n = 6 (*myd88*^+/−^ control), n = 5 (*myd88*^+/−^ *cxcl18b* OE), n = 6 (*myd88*^*−/−*^ control), and n = 5 (*myd88*^*−/−*^ *cxcl18b* OE). Experimental plan in Fig. 6d. **e**, Genomic sequence of the *cxcl18b*^*bns683*^ full locus deletion allele. **f**, Experimental plan and RT-qPCR analysis of *cxcl18b* mRNA levels in *cxcl18b*^+/+^ and *cxcl18b*^*−/−*^ larvae 6 hours post fin fold amputation (hpa). The dots in the graph represent individual larvae; data are shown as the mean ± s.d.; n = 4 *cxcl18b*^+/+^ and n = 3 *cxcl18b*^*−/−*^. Statistical test: Student’s *t*-test. Ct values listed in Supplementary Table 3. Yellow dashed lines delineate the injured area. Scale bars, 100 μm. Source data

To gain a deeper understanding of the targets of the MyD88 signaling pathway during zebrafish cardiac regeneration, we conducted bulk RNA-seq analysis on *myd88*^−/−^ and *myd88*^+/+^ untouched (UT) ventricles and injured tissues at 1 and 24 hpci (Fig. 6a). As anticipated, we observed decreased expression of several immune-related genes in cryoinjured *myd88*^−/−^ tissues, including *cxcl18b* (Fig. 6b). Through cross-examination of our transcriptomic datasets with publicly available datasets, we found that *cxcl18b* was significantly upregulated (*P*_adj_ < 0.05 and FDR > 1) at early time points after cardiac injury in WT zebrafish^10,81,82^. *cxcl18b* is also upregulated in zebrafish after infection and fin amputation, where it regulates neutrophil recruitment^32,83–85^. Importantly, a previous study reported that *cxcl18b* upregulation is downstream of MyD88 signaling in infection models^32^; however, the specific role of Cxcl18b during cardiac regeneration remains to be elucidated. Regarding its expression pattern, *cxcl18b* is expressed in caudal hematopoietic and endothelial cells in infected zebrafish larvae^83^ and in endocardial cells in regenerating zebrafish hearts^34^. We used a *Tg(cxcl18b:EGFP)* reporter line^83^ to look more closely at *cxcl18b* expression and observed clear EGFP expression in endocardial and epicardial cells at 24 hpci (Extended Data Fig. 10a).

We next investigated *cxcl18b* expression using real-time quantitative polymerase chain reaction (RT–qPCR) and observed clear downregulation in cryoinjured *myd88*^−/−^ ventricles compared with *myd88*^+/+^ siblings (Fig. 6c). We then developed gain-of-function and LOF genetic tools to investigate Cxcl18b function during heart regeneration. As Cxcl18b is a chemokine that attracts neutrophils, we used the gain-of-function tool to determine whether *cxcl18b* overexpression could restore the low neutrophil levels observed in cryoinjured *myd88*^−/−^ ventricles (Fig. 1d,f,g). For this purpose, we generated a *Tg(hsp70l:LBL-cxcl18b-t2a-mCherry)* line, which allows for conditional overexpression of *cxcl18b* under the control of the *hsp70l* promoter upon Cre-mediated recombination using the HOTcre system^86^. To achieve global *cxcl18b* overexpression, we injected transgenic embryos with *Cre* mRNA and performed heat shocks (Extended Data Fig. 10b). Successful overexpression of *cxcl18b* and *mCherry* was observed using RT–qPCR (Extended Data Fig. 10c). We raised *Cre* mRNA-injected (*cxcl18b* overexpression) *myd88*^+/−^ and *myd88*^−/−^ zebrafish and uninjected (control) *myd88*^+/−^ and *myd88*^−/−^ *Tg(hsp70l:LBL-cxcl18b-t2a-mCherry); TgBAC(mpx:GFP)* siblings to adulthood. We then performed heat shocks and cryoinjury and quantified the number of *mpx:*GFP^+^ cells at 24 hpci (Fig. 6d). *Cre* mRNA-mediated recombination was validated by the detection of the recombination marker, mCherry (Extended Data Fig. 10d). While the number of *mpx:*GFP^+^ cells was reduced in control cryoinjured *myd88*^−/−^ ventricles, when we overexpressed *cxcl18b*, there was no significant difference in neutrophil numbers between *myd88*^−/−^ and *myd88*^+/−^ samples, indicating that *cxcl18b* overexpression is sufficient to at least partially rescue the low neutrophil numbers observed in cryoinjured *myd88*^−/−^ ventricles (Fig. 6e,f).

To understand how loss of Cxcl18b might affect neutrophil recruitment during cardiac regeneration, we generated a *cxcl18b* mutant allele using the CRISPR–Cas9 technology. As many chemokines have high sequence homology and to avoid transcriptional adaptation events^87,88^, we specifically generated a full locus deletion allele (Extended Data Fig. 10e). To assess *cxcl18b* expression levels, we performed larval fin fold amputations, which trigger *cxcl18b* upregulation in WT cells^84,85^, and collected larvae 6 hours post amputation (hpa) for RT–qPCR analysis. As anticipated, *cxcl18b* full locus deletion mutants completely lacked *cxcl18b* expression (Extended Data Fig. 10f). To examine whether lack of Cxcl18b could affect neutrophil recruitment in cryoinjured ventricles, we quantified neutrophil abundance at 24 and 96 hpci. Notably, while the Mpx^+^ cell count was unaffected at 24 hpci, there was a significant difference between *cxcl18b*^−/−^ and *cxcl18b*^+/+^ siblings at 96 hpci (Fig. 6g,h).

In summary, these findings together indicate that Cxcl18b is a chemokine whose expression is upregulated upon cardiac cryoinjury by MyD88 and is at least partly responsible for neutrophil recruitment to the injured area.

## Discussion

**Fig. 7:**
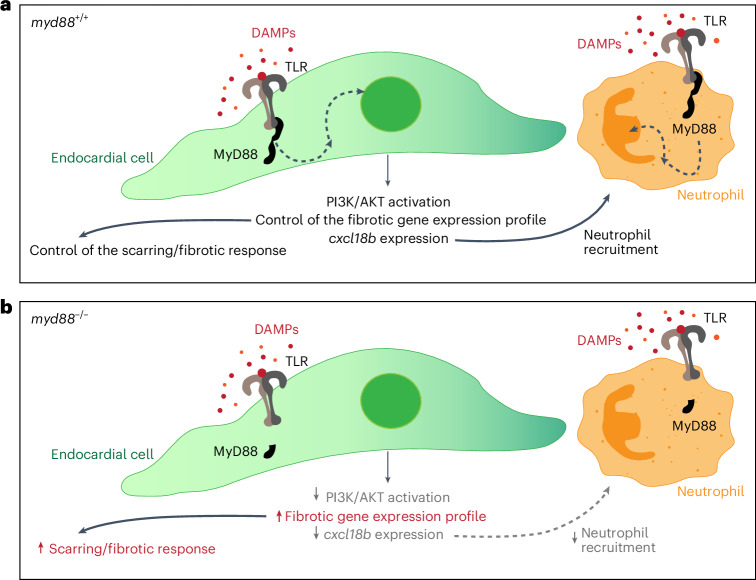
Proposed model for the role of MyD88 in fibrosis and neutrophil recruitment following cardiac cryoinjury in zebrafish. **a**, TLRs (and IL-1Rs) recruit the adaptor molecule MyD88 upon ligand interaction. MyD88 in turn initiates signal transduction, which leads to the activation of several processes. In endocardial cells, PI3K/AKT pathway activation and fibrosis are controlled by the MyD88 signaling pathway. Neutrophil count is also partially affected by the levels of the endocardial chemokine Cxcl18b. Immune cells also activate a MyD88-mediated response leading to several processes, including the recruitment of more immune cells and the manifestation of an inflammatory response. **b**, In *myd88*^−/−^ injured tissues, the MyD88 signaling pathway is not activated and thus, endocardial cells exhibit decreased activation of the PI3K/AKT pathway. *myd88*^−/−^ injured tissues also exhibit decreased levels of the endocardial chemokine gene *cxcl18b* and an increase in features related to fibrosis. In addition, the neutrophil count appears significantly reduced. As the fibrotic and the immune responses are affected, other processes essential for successful regeneration are also impaired, including revascularization and CM proliferation. Source data

After cardiac injury, the zebrafish heart mounts a robust innate immune response, which is crucial for the regeneration process^9–11^. The precise regulation of this early immune response is essential, underscoring the need for a detailed understanding of its regulators and underlying mechanisms^14^. Key activators of this response are the TLR and IL-1R signaling pathways, which signal predominantly through the adapter protein MyD88 (refs. ^16–19^). While previous studies mostly focused on immune cells to investigate MyD88 function, in this study we examined other cell types of the heart and identified an unexplored function for MyD88: not only does it regulate the inflammatory response, but it also limits the endocardial fibrotic response to injury. Specifically, we observed a significant reduction in pro-inflammatory neutrophil and macrophage populations in cryoinjured *myd88*^−/−^ ventricles. More surprisingly, we also observed in *myd88*^−/−^ ventricles (1) the expansion of a collagen-rich endocardial population, (2) compromised endocardial PI3K/AKT pathway activation, (3) an increased number of myofibroblasts and (4) increased fibrin levels, as well as bigger scars. Our data further revealed that lack of MyD88 signaling impairs CM behavior in the injured area. By endothelial cell-specific overexpression of *myd88*, we showed that it has an essential role in the endothelial response to injury by regulating neutrophil recruitment to the injury site as well as fibrosis. Mechanistically, we identified the chemokine gene *cxcl18b* as a target of the MyD88 signaling pathway that controls neutrophil recruitment (Fig. 7). Overall, these data highlight the beneficial role of MyD88 activation in the regenerative response to cardiac injury in zebrafish and provide insights on how pathways activated very quickly after injury can shape the regenerative outcome.

While some studies suggested that MyD88 signaling promotes the regenerative process, others reported improved outcomes when MyD88 signaling was blocked^14,20,21^. Our data clearly show that MyD88 is a beneficial molecule for the regenerative process in zebrafish. The discrepancy with studies reporting detrimental effects of MyD88 signaling on regeneration mostly lies in the model used. Specifically, while inflammation is essential in the early stages of regeneration^9–11^, prolonged and excessive inflammation can lead to adverse outcomes^14,20^. In non-regenerative mouse models, inflammation tends to be uncontained; thus, limiting inflammation by blocking MyD88 signaling can enhance the regeneration potential^22–24,89–91^. Accordingly, blocking MyD88 function has been linked with protective effects in experimental settings (for example, endotoxin shock) after lipopolysaccharide administration^23,92,93^. Once again, these data reflect special cases where inflammation is uncontrolled and excessive.

In addition, following *myd88* overexpression in endothelial cells, we successfully reversed the reduced neutrophil number and the enhanced fibrosis phenotypes observed in cryoinjured *myd88*^−/−^ ventricles, indicating that MyD88 is required in endothelial cells for the regenerative response. However, *myd88* overexpression in endothelial cells in cryoinjured *myd88*^+/+^ ventricles did not lead to an increase in neutrophil numbers (Figs. 1g and 5h) or an improvement in the fibrotic response (Figs. 2c–g and 5a–f), indicating that MyD88 signaling is optimally tuned in WT conditions. Studies reported that endothelial-specific MyD88-deficient mice exhibit features of decreased inflammation^94^, while stimulation of the MyD88 pathway in aortic endothelial cells in rabbits promoted inflammation^95^. In light of our data, it will be interesting to investigate in injured mammalian hearts which cells activate MyD88 signaling and the function of the MyD88 pathway in endocardial cells.

Myeloid cells serve as key activators of the MyD88 signaling pathway in response to injury or infection^22,37–40^. The *myd88* LOF mutant used in this study has also been used in the context of larval zebrafish tail fin regeneration, where it was reported that MyD88 controls neutrophil and macrophage recruitment to the wound^37^. In line with these findings, we observed reduced neutrophil and macrophage numbers in cryoinjured *myd88*^−/−^ ventricles. However, we do not anticipate major differences between *myd88*^−/−^ and WT zebrafish in the absence of an insult (injury or infection) that could trigger MyD88 pathway activation. As shown previously, early leukocyte hematopoiesis, migration, basal motility and phagocytosis are not affected by MyD88 deficiency^32,37^. In addition, transcriptomic analysis from uninfected *myd88*^−/−^ and WT embryos did not reveal any differences in immune-related genes other than *myd88*, which was explained by the lower stability of the mutant transcript^32^. In our study, we also did not observe any significant differences in neutrophil count between sham-injured *myd88*^−/−^ and *myd88*^+/+^ ventricles (Extended Data Fig. 1f,g). Altogether, these data indicate that the observed phenotypes in cryoinjured *myd88*^−/−^ ventricles are due to the mutant’s inability to activate the pathway in response to injury rather than to preexisting developmental defects.

We expanded our investigation beyond the conventional activators of the MyD88 signaling pathway, myeloid cells, to explore the impact of the loss of MyD88 function on other cell types. Our transcriptomic analysis pointed to endocardial cells, which in *myd88*^−/−^ ventricles display a fibrotic phenotype. Bulk RNA-seq analysis of endocardial cells revealed impairment in the activation of the MAPK and PI3K/AKT pathways in the *myd88*^−/−^ endocardium. We elected to focus on MyD88 function in the PI3K/AKT pathway because it was more strongly affected and has not been explored as extensively as the MAPK pathway in the endocardial response during cardiac regeneration in zebrafish^51,63,64^. For these and other studies, we then overexpressed *myd88* in endothelial cells in cryoinjured *myd88*^−/−^ ventricles, which led to the reversal of the reduced neutrophil count and the enhanced fibrosis. Taken together, these findings underscore the importance of the MyD88 signaling pathway in endocardial cells and thus support the hypothesis that at least some of the *myd88*^−/−^ phenotypes after cardiac injury could originate mostly in the endocardium. Unlike most myeloid cells, which need to be recruited to the injured tissue, endocardial cells are inherently present and abundant in the heart^36,96^. Additionally, a previous study showed that MyD88 signaling in hematopoietic cells alone was not sufficient to trigger the inflammatory response in the injured mouse heart^97^. Specifically, myocardial infarction in *Myd88*^−/−^ and WT mice reconstituted with WT bone marrow cells led to no differences in cytokine or chemokine levels^97^. Furthermore, endothelial cells activate immune-regulatory programs, secrete cytokines and chemokines, and initiate immune cell recruitment^52,55,80^. They express the *tlr* and *myd88* genes^52,53^ and at least a subgroup of endothelial cells, vascular endothelial cells, rely exclusively on MyD88 for TLR activation^98^. Additionally, signaling cascades commonly activated by MyD88, such as nuclear factor kappa-light-chain-enhancer of activated B cells (NF-κB) and PI3K/AKT, get activated in endothelial cells^69,99^. These studies, together with our data, emphasize the pivotal role of early MyD88 signaling activation in endocardial cells and support the hypothesis that the fibrotic phenotype in cryoinjured *myd88*^−/−^ ventricles initiates in the endocardium.

During cardiac development and regeneration, the endocardium has a crucial role in signaling to CMs^55,100^, while the ECM supports CM behavior^101–103^. Given the altered inflammatory and fibrotic environment observed in cryoinjured *myd88*^−/−^ ventricles, along with the impaired endocardial response preceding CM appearance in the injured area, we investigated CM behavior. Our findings revealed decreased CM proliferation and reduced CM protrusion length in cryoinjured *myd88*^−/−^ ventricles. We hypothesize that the *myd88*^−/−^ CM environment is not sufficiently permissive for CM protrusions to extend as they do in *myd88*^+/+^ siblings. However, we cannot exclude the possibility that CMs also activate MyD88 signaling, as activity of NF-κB, a MyD88 target, has also been reported in CMs^104^. Unfortunately, our scRNA-seq analysis did not manage to capture a CM population; similar challenges with CM populations have been reported in other transcriptomic studies^36,105^. In addition, it is probable that epicardial cells also activate MyD88 signaling during cardiac regeneration in zebrafish. The number of superficially localized αSMA^+^ cells, presumably of epicardial, fibroblast and perivascular origin, was also elevated in cryoinjured *myd88*^−/−^ ventricles (Extended Data Fig. 2e). To draw more definitive conclusions about the role of MyD88 in CMs, epicardial cells and other cardiac cell populations, additional cell type-specific studies will need to be performed.

Our data also reveal that the expression of Cxcl18b, a neutrophil chemoattractant produced in the endocardium, is induced after MyD88 activation. Notably, we observed that *cxcl18b* overexpression partially rescued the reduced neutrophil count in cryoinjured *myd88*^−/−^ ventricles (Fig. 6e,f). Neutrophil recruitment via Cxcl18b was previously reported not to be dependent on the dosage of Cxcl18b because receptor saturation might occur or neutrophils might not be able to get stimulated further^83^. Our inability to fully rescue neutrophil numbers with *cxcl18b* overexpression might, at least in part, be due to these issues or the involvement of other chemoattractants. We also generated a *cxcl18b* full locus deletion allele and showed that homozygous mutants displayed significantly reduced neutrophil numbers at 96 hpci. As there was no clear difference in neutrophil count at 24 hpci between cryoinjured *cxcl18b*^−/−^ and *cxcl18b*^+/+^ ventricles, we again speculate that Cxcl18b is one of several chemokines secreted after injury and required for neutrophil recruitment^83^, and that its absence alone is not sufficient to trigger a difference at early time points.

In summary, our findings highlight the crucial role of MyD88 signaling in cardiac regeneration. We propose that MyD88 regulates both the inflammatory and fibrotic responses and that its early activation in the endocardium is vital for successful regeneration. Additionally, we identified the chemokine Cxcl18b as a target of MyD88 signaling that contributes to neutrophil recruitment. Gaining a deeper understanding of the early innate immune mechanisms activated in the injured heart is of vital importance because they can influence subsequent processes and thereby impact the regenerative outcome. In addition, a comprehensive understanding of the MyD88 pathway’s contribution to the regenerative response will determine whether targeting a molecule of the innate immune system could be considered as a treatment target to limit pathogenesis in cardiovascular diseases.

## Methods

### Zebrafish husbandry and handling

Zebrafish larvae were raised under standard conditions. Adult fish were maintained in 3.5-l tanks at a stock density of ten fish per liter with the following parameters: water temperature, 27–27.5 °C; light–dark cycle, 14:10; pH, 7.0–7.5; conductivity, 750–800 µS cm^−2^. Zebrafish were fed 3–5 times a day, depending on age, with granular and live food (*Artemia salina*). Health monitoring was performed twice a year. All procedures performed on animals conformed to the guidelines from Directive 2010/63/EU of the European Parliament on the protection of animals used for scientific purposes and were approved by the Animal Protection Committee (Tierschutzkommission) of the Regierungspräsidium Darmstadt (reference nos. B2/1218 and B2/1229).

### Zebrafish lines

The following mutant and transgenic lines were used: *myd88*^*hu3568*^ (ref. ^32^), *cxcl18b*^*bns683*^ (this study), *TgBAC(mpx:GFP)*^*i114*^ (ref. ^106^), *Tg(mpeg1:EGFP)*^*gl22*^ (ref. ^107^), *ET(krt4:EGFP)*^*sqet33-1A*^ ref. ^108^, abbreviated as *ET(krt4:EGFP)*, *Tg(-0.8flt1:RFP)*^*hu5333*^ (ref. ^109^), *Tg(fli1a:myd88,EGFP)*^*bns703*^ (this study), *Tg(cxcl18b:EGFP)*^*ibl150*^ (ref. ^83^) and *Tg(hsp70l:loxP-TagBFP-loxP-cxcl18b-t2a-mCherry)*^*bns660*^ (this study), abbreviated as *Tg(hsp70l:LBL-cxcl18b-t2a-mCherry)*.

### Generation of zebrafish mutant and transgenic lines

Sequence analysis for the generation of new lines was performed using the ApE software (v.2.0.61). To generate the *cxcl18b*^*bns683*^ full locus deletion allele, the CRISPR–Cas9 technology was used, as described previously^110–112^. The single-guide RNAs (sgRNAs) GGAAAGTTACAAGGAAATGC and GGAAGTTTGGATGATTCTAA were designed with a CRISPR design tool (https://www.crisprscan.org/) targeting sequences upstream and downstream of the 5′ and 3′ untranslated regions, respectively. sgRNAs were transcribed using a MEGAshortscript T7 Kit (Thermo Fisher Scientific) and later purified with the RNA Clean & Concentrator Kit (Zymo Research). Then, 50 pg of each sgRNA were coinjected with 100 pg of *cas9* mRNA into zebrafish one-cell-stage WT embryos. Injected embryos were raised to adulthood and later screened for founder identification. To detect the full locus deletion allele, we performed PCR using the forward 5′-GTACCCTGGTTAATGACTAATCCTAGTT-3′ and reverse 5′-AGCTGATCAGAACGCACAGTAACG-3′ primers, leading to a 310-bp product. To detect the WT allele, we performed PCR using the forward 5′- ATCCACAAAAACAGCAGGGC-3′ and reverse 5′- CATCTTCAGCGAGTCGGTGT-3′ primers, leading to a 739-bp product.

To generate the *Tg(fli1a:myd88,EGFP)*^*bns703*^ line, in which a bidirectional *fli1a* promoter drives the expression of both *myd88* and *EGFP*, we modified the *fli1a:mCherry,EGFP* construct provided by C. Helker. The *myd88* coding sequence was cloned and subsequently used to replace the *mCherry* coding sequence. Cloning was performed using the In-Fusion HD Cloning Kit (Takara Bio). Then, 10 pg of the final construct were coinjected with 25 pg of *Tol2* mRNA into zebrafish one-cell-stage WT embryos. Injected embryos positive for EGFP were raised to adulthood and later screened for founder identification.

To generate the *Tg(hsp70l:loxP-TagBFP-loxP-cxcl18b-t2a-mCherry)*^*bns660*^ line, the *hsp70l:loxP-TagBFP-loxP-il11ra*-*t2a*-*mCherry* construct was modified^59^. The *cxcl18b* coding sequence was cloned and subsequently used to replace the *il11ra* coding sequence. Cloning was performed using the In-Fusion HD Cloning Kit. Then, 10 pg of the final construct were coinjected with 25 pg of *Tol2* mRNA into zebrafish one-cell-stage WT embryos. Injected embryos were heat-shocked and embryos positive for TagBFP were raised to adulthood and later screened for founder identification. To induce recombination, 12.5 pg of *Cre* mRNA were injected into *Tg(hsp70l:loxP-TagBFP-loxP-cxcl18b-t2a-mCherry)* one-cell-stage embryos. *Cre* mRNA-injected embryos were heat-shocked and embryos positive for *mCherry* were raised to adulthood.

### Cardiac cryoinjury and heat shock treatments

Cardiac cryoinjury was performed in adult zebrafish hearts as described previously^3–5^. Zebrafish were briefly anesthetized with tricaine and transferred on a wet sponge with their ventral side up. A small incision was made on the chest area exposing the heart. A cryoprobe precooled in liquid nitrogen was applied to the ventricular apex until thawing. Cryoinjured fish were transferred into fresh system water and left to recover. Heat shock treatments to induce the expression of the *hsp70l*-driven transgene *Tg(hsp70l:loxP-TagBFP-loxP-cxcl18b-t2a-mCherry)* were performed by incubating the adult fish or the embryos in preheated system or egg water (39 °C) for 1 h. Several heat shocks were performed as described in the corresponding experimental plans. It is important to note that heat shock alone, as a stress response, can promote an immune response, including the influx of inflammatory cells^113,114^, potentially explaining the discrepancy in neutrophil count in cryoinjured ventricles observed with (Fig. 6f) and without (Fig. 1g) heat shock treatments.

### Histological analysis and imaging

For the histological analysis, zebrafish hearts were fixed in 4% paraformaldehyde for 1 h at room temperature and then preserved overnight in 30% (w/v) sucrose solution prepared in 1× PBS at 4 °C. Hearts samples were embedded in O.C.T. (Tissue-Tek) and stored at −80 °C until further use. Eight and 50-μm-thick cryosections were collected on SuperFrost Plus slides (Thermo Fisher Scientific) using the Leica CM1950 cryostat and stored at −20 °C.

For AFOG staining, slides were thawed for 15 min at room temperature, rinsed twice with 0.1% Triton X-100 in 1× PBS to remove O.C.T., incubated in Bouin’s solution for 2 h at 60 °C and then stained according to the manufacturer’s instructions (AFOG staining kit, BioGnost) and without hematoxylin solution^59^. Stained slides were imaged using a Nikon SMZ25 stereo microscope coupled with a Nikon Digital Sight DS-Ri1 camera.

For immunofluorescence staining, slides were thawed for 15 min at room temperature, rinsed twice with 0.1% Triton X-100 in 1× PBS to remove O.C.T. and permeabilized with 0.5% Triton X-100 in 1× PBS for 20 min (2 h for 50-μm-thick cryosections) at room temperature. Cryosections were then incubated in blocking buffer solution (1× PBS, 2% (v/v) goat serum, 0.2% Triton X-100 and 1% dimethyl sulfoxide) for 1 h at room temperature. Then, cryosections were incubated with primary antibodies in blocking buffer solution overnight at 4 °C, rinsed three times every 10 min with 0.1% Triton X-100 in 1× PBS and incubated with secondary antibodies in blocking buffer solution for 3 h at room temperature. Lastly, immunostained cryosections were rinsed three times every 10 min with 0.1% Triton X-100 in 1× PBS, incubated in DAPI (1:10,000 dilution, Sigma-Aldrich) for 5 min at room temperature and mounted with fluorescence mounting medium (cat. no. S3023, Agilent Dako) for imaging. MEF2, PCNA, pAkt and pERK immunostaining was performed as described previously^73^. MEF2 and pERK immunostaining was supplemented with an additional step after O.C.T. removal, which consisted of antigen retrieval in 10 mM sodium citrate buffer with 0.05% (v/v) Tween-20 (all from Sigma-Aldrich), pH 6.0, for 7 min at 95 °C.

Primary antibodies used were: anti-GFP at 1:500 dilution (chicken, cat. no. GFP-1010, Aves Labs); anti-αSMA at 1:200 dilution (rabbit, cat. no. GTX124505, GeneTex); anti-Fli1 (clone EPR4646) at 1:100 dilution (rabbit, cat. no. ab133485, Abcam); anti-pAkt (clone 6F5) at 1:200 dilution (mouse, cat. no. 05-1003, Sigma-Aldrich); anti-PCNA (clone PC10) at 1:200 dilution (mouse, cat. no. sc-56, Santa Cruz Biotechnology); anti-MEF2 at 1:100 dilution (rabbit, cat. no. DZ01398, Boster Bio); anti-Mpx at 1:200 dilution (rabbit, cat. no. GTX128379, GeneTex); anti-Aldh1a2 (clone G-2) at 1:100 dilution (mouse, cat. no. sc-393204, Santa Cruz Biotechnology); anti-Aldh1a2 at 1:200 dilution (rabbit, cat. no. GTX124302, GeneTex); anti-zf-Cdh5 at 1:100 dilution (rabbit, cat. no. AS-55715, AnaSpec); anti-pERK (clone D13.14.4E) at 1:100 dilution (rabbit, cat. no. 4370S, Cell Signaling Technology); anti-RFP at 1:200 dilution (rabbit, cat. no. 600-401-379, Rockland Immunochemicals); N2.261 at 1:20 dilution (mouse, developed by H. M. Blau and obtained from the Developmental Studies Hybridoma Bank); and anti-DsRed at 1:200 dilution (recognizing mCherry, Living Colors, rabbit, cat. no. 632496, Takara Bio). Secondary antibodies used (all at 1:500 dilution) were: anti-chicken IgG (H+L) Alexa Fluor 488 (goat, cat. no. A-11039, Invitrogen); anti-mouse IgG (H+L) Alexa Fluor 488 (goat, cat. no. A-11029, Invitrogen); anti-mouse IgG (H+L) Alexa Fluor 568 (goat, cat. no. A-11004, Invitrogen); and anti-rabbit IgG (H+L) Alexa Fluor 647 (goat, cat. no. A-21244, Invitrogen). Phalloidin-Alexa Fluor 568 (cat. no. A12380, Thermo Fisher Scientific) was used at a 1:200 dilution. Imaging was performed using a ZEISS LSM 800 Observer inverted confocal microscope, a ZEISS Cell Observer Spinning Disk inverted confocal microscope, a ZEISS Axioscan 7 microscope, a Nikon Ni-E Eclipse widefield microscope equipped with a SlideExpress 2 slideloader (Märzhäuser), a SOLA Light Engine (Lumencor) and a DS-Qi2 Mono Digital Microscope Camera (Nikon). For the ZEISS and Nikon microscopes, the ZEN Blue Edition and NIS-AR software v.5.3 were used, respectively. Wholemount ventricle imaging was performed using a Nikon SMZ25 microscope coupled with a Nikon Digital Sight DS-Ri1 camera and the NIS-Elements v.4.30 software.

### Quantification and statistical analysis

Quantification was done in two or three non-consecutive sections per ventricle and with the ZEN Blue Edition software. Quantification of the *mpx*:GFP^+^, *mpeg1*:EGFP^+^ and Mpx^+^ cell numbers was performed in the injured tissue and in peripheral border zone areas (100 μm). The *mpx*:GFP^+^ cell number analysis for the *myd88*^+/−^ control condition (Fig. 6e,f) was performed separately. Quantification of the total αSMA^+^ cell number was performed in the injured tissue, and of the intraventricular αSMA^+^ cell number was performed in the injured tissue excluding the superficial/peripheral αSMA^+^ cells (that is, quantification was based on cell localization and not on the presence of an additional cell marker). Quantification of intraventricularly localized αSMA^+^ cells did not include double-positive αSMA^+^ *krt4*:EGFP^+^ cells for the following reasons: (1) the endocardium has not fully extended into the injured tissue at 96 hpci (the time point of our analysis), as indicated by *krt4*:EGFP^+^ expression, and (2) even though αSMA^+^ cells can derive from the endocardium, it is not known how long they retain the expression of endocardial markers. The αSMA^+^ cell number analysis displayed in Fig. 2d is the result of two independent experiments, each of which contained samples of both genotypes from the same batch. For the endocardial pAkt activation and proliferation analysis, the percentage of pAkt^+^ and PCNA^+^ endocardial cells was calculated as a ratio of the total number of endocardial cells (*krt4*:EGFP^+^Fli1^+^) in a 50-μm wide area on the basal-most side of the injured tissue, as described previously^55^. For the *krt4*:EGFP^+^, Cdh5^+^ and pERK^+^ cell area analysis, the fluorescent area within the injured tissue was measured and then divided by the total injured tissue area. For the CM dedifferentiation and proliferation analysis, the percentage of N2.261^+^ and PCNA^+^ CMs was calculated as a ratio of the total number of CMs (MEF2^+^) in peripheral border zone areas (100 μm). CM protrusions and protrusion lengths were measured in two non-consecutive 50-μm-thick cryosections with the largest injured area from each heart. The start of the protrusions was defined as the injury border and the end of the protrusions as the point where the F-actin signal disappeared in the injured tissue, as described previously^77^. All protrusions measured were extending toward the injured tissue. For the cEC proliferation analysis, the percentage of PCNA^+^ cECs was calculated as a ratio of the total number of cECs (*-0.8flt1*:RFP^+^) in the injured tissue and in peripheral border zone areas (200 μm). For the coronary vessel coverage analysis, the percentage of the fluorescence intensity was calculated as a ratio of the background fluorescence in the injured tissue using ImageJ (v.1.53c) in wholemount images. For the scar area analyses, scar areas were selected based on the combined occurrence of collagen and fibrin within the ventricle. The average of the ratio of the 2–3 biggest scar areas to the total ventricular areas were calculated using ImageJ (v.1.53c). Percentages of scar areas (relative to ventricular areas) were then grouped based on their size. Fibrin and collagen measurements were performed as described previously^62^.

All statistical analyses were performed in GraphPad Prism (v.9). Distribution of data in each group was assessed using the Shapiro–Wilk normality test. Data that were normally distributed were further analyzed with a two-tailed Student’s *t*-test. Data that were not normally distributed were further analyzed with a two-tailed Mann–Whitney *U*-test. The significance level was set to 0.05 for all tests. The exact *P* values are indicated in the figures. The error bars in the figures represent the mean ± s.d.

### Tissue dissociation and cell sorting

For the scRNA-seq experiment, cardiac cells were isolated from a pool of four *myd88*^+/+^ and four *myd88*^−/−^ ventricles, and for the endocardial bulk RNA-seq experiment from a pool of four *ET(krt4:EGFP); myd88*^+/+^ and four *ET(krt4:EGFP); myd88*^−/−^ ventricles for each sample. Cell isolation was performed according to the manufacturer’s instructions (Pierce Primary Cardiomyocyte Isolation Kit, cat. no. 88281, Thermo Fisher Scientific) and with the following modifications: incubation was performed at 30 °C for 20 min, followed by resuspension in 1× Hanks’ Balanced Salt Solution (cat. no. 14175053, Gibco) with 0.25% BSA. The cell suspension was passed through a round bottom polystyrene test tube fitted with a 35-µm nylon mesh filter cap (Falcon, cat. no. 352235, Corning). DAPI (cat. no. D954, Sigma-Aldrich) was added before sorting. For the scRNA-seq experiment, resuspended cells were sorted using a BD FACSAria III Cell Sorter (BD Biosciences) equipped with a 100-µm nozzle and at an instrument pressure setting of 20 psi. Dead cells were excluded using DAPI excited by a 30-mW 405-nm laser paired with a 450/50-nm band-pass filter. For the endocardial bulk RNA-seq experiment, resuspended cells were sorted using an Invitrogen Bigfoot Spectral Cell Sorter (Thermo Fisher Scientific) equipped with a 100-µm nozzle tip and at an instrument pressure setting of 20 psi. Dead cells were excluded using DAPI excited by a 100-mW 355-nm laser paired with 455/14-nm band-pass filter. EGFP fluorescence was measured with a 100-mW 488-nm excitation paired with a 530/30-nm band-pass filter. Sorted EGFP^+^ cells were resuspended in ice-cold QIAzol Lysis Reagent (QIAGEN), flash-frozen in liquid nitrogen and kept at −80 °C until RNA extraction. Cytometric data were recorded using the FACSDiva software v.8.0.1 (BD Biosciences) and the Sasquatch software v.1.19.2 (Thermo Fisher Scientific). The .fcs files were analyzed using FlowJo v.10.8.1 (BD Life Sciences).

### scRNA-seq analysis

For the scRNA-seq experiment, cells were counted with a Moxi cell counter and diluted according to the manufacturer’s protocol to obtain 10,000 single-cell data points per sample. Each sample was run separately on a lane in a Chromium controller with the Chromium Next GEM Single Cell 3′ Reagent Kits v.3.1 (10x Genomics). scRNA-seq library preparation was done using a standard protocol. Sequencing was done on NextSeq 2000 system and raw reads were aligned against the zebrafish genome (DanRer11) and counted using STARsolo^115^ followed by secondary analysis in annotated data format. Preprocessed counts were further analyzed using Scanpy^116^. Basic cell quality control was conducted by taking the number of detected genes and mitochondrial content into consideration. We removed 32 cells that did not express more than 300 genes or had a mitochondrial content greater than 10%. Furthermore, we filtered 7,823 genes if they were detected in fewer than 30 cells (<0.01%). The raw counts per cell were normalized to the median count over all cells and transformed into log space to stabilize variance. We initially reduced the dimensionality of the dataset using principal component analysis, retaining 50 principal components. Final data visualization was done using the scVelo^117,118^ and CELLxGENE packages.

### Bulk RNA-seq analysis

For bulk RNA-seq of endocardial cells (Fig. 3a), RNA was isolated from the fluorescence-activated cell-sorted endocardial cells using the miRNeasy Micro Kit (QIAGEN) combined with on-column DNase digestion (RNase-free DNase Set, QIAGEN) to avoid contamination by genomic DNA (gDNA). Subsequent RNA quality control analysis, complementary DNA (cDNA) preparation and sequencing were performed by Novogene. mRNA was purified from total RNA with poly-T oligo-attached magnetic beads, went through fragmentation and cDNA was synthesized. Trimmomatic^119^ was used to trim the reads. Reads longer than 15 nucleotides after trimming were kept and aligned to the Ensembl zebrafish genome v.danRer11 (Ensembl release 109) with STAR aligner (v.2.7.10a)^115^. Alignments were filtered with the Picard tool (v.3.0.0) to remove duplicates, multimapping, ribosomal or mitochondrial reads. Gene counts were generated using the featureCounts tool (v.2.0.4), taking all reads overlapping annotated exons into account and excluding those overlapping multiple genes^120^. Finally, the raw count matrix was normalized and contrasts were created and analyzed using DESeq2 (v.1.36.0)^121^. Genes were classified as significantly differentially expressed at an average count greater than five, multiple testing *P*_adj_ < 0.05 and −0.585 < log_2_ fold change > 0.585. Heatmaps with DEGs were obtained using the WIlsON^122^. To perform GSEA, we used the Python package gseapy^123^. As gene sets, we used 162 custom *Danio rerio* mapped gene sets from reactome and 3,023 gene sets from Gene Ontology. Gene ranking was performed according to the DESeq-derived *P*_adj_ multiplied by the direction (+ or −) of the log_2_ fold change. The GSEA analysis was run for gene sets with a min_size = 5, max_size = 1,000 and permutation_num = 1,000. From the resulting lists, representative sets were selected.

For bulk RNA-seq of untouched ventricles and injured tissues (Fig. 6a), RNA was isolated from three pooled ventricles or three pooled injured tissues per sample using the miRNeasy Micro Kit (QIAGEN) combined with on-column DNase digestion (RNase-free DNase Set, QIAGEN) to avoid contamination by gDNA. Two biological replicates were prepared for each condition. RNA and library preparation integrity were verified with LabChip Gx Touch 24 Analyzer (PerkinElmer). 400 ng of total RNA was used as input for the VAHTS Stranded mRNA-seq Library Preparation V6 according to the manufacturer’s protocol (Vazyme). Sequencing was performed on an NextSeq 500 instrument (Illumina) using v2 chemistry with 1 × 75-bp single end setup. Trimmomatic v.0.39 was used to trim reads after a quality drop below a mean of Q15 in a window of five nucleotides, keeping only filtered reads longer than 15 nucleotides^119^. Reads were aligned against the Ensembl zebrafish genome v.danRer11 (Ensembl release 99) with STAR v.2.7.3a^115^. Alignments were filtered with Picard v.2.21.7 to remove duplicates, multimapping, ribosomal or mitochondrial reads. Gene counts were established with featureCounts v.1.6.5 aggregating reads overlapping exons, excluding those overlapping multiple genes^120^. The raw count matrix was normalized with DESeq2 v.1.26.0 (ref. ^121^). Contrasts were created with DESeq2 based on the raw count matrix. Genes were classified as significantly differentially expressed at average count greater than five, multiple testing *P*_adj_ < 0.05 and −0.585 < log_2_ fold change > 0.585. Heatmaps with DEGs were obtained using WIlsON^122^.

### RT–qPCR

For RT–qPCR, RNA was extracted from three pooled cryoinjured tissues (1 hpci) and ventricles (96 hpci) (Fig. 6c) or from single larvae (Extended Data Fig. 9a and Extended Data Fig. 10c,f) or from single cryoinjured ventricles (96 hpci) (Extended Data Fig. 9a) per biological replicate using the TRIzol Reagent (Thermo Fisher Scientific) using the phenol–chloroform extraction protocol. Total RNA was purified with the RNA Clean & Concentrator Extraction Kit (Zymo Research) according to the manufacturer’s instructions. At least 250 ng of total RNA per sample was reverse-transcribed with the Maxima First Strand cDNA Synthesis Kit (Thermo Fisher Scientific) according to the manufacturer’s instructions. All reactions were performed with three technical replicates using the SYBR Green PCR Master Mix (Thermo Fisher Scientific) on the CFX Connect Real-Time System (CFX Manager 3.1, Bio-Rad Laboratories) and with the following program: preamplification at 95 °C for 7 min followed by 39 cycles of amplification at 95 °C for 5 s and 60 °C for 20 s, using a melting curve from 60 to 92 °C with an increment of 1.0 °C every 5 s. Gene mRNA levels were normalized against the *rpl13a* mRNA levels and fold changes were calculated using the $2-^{\Delta\Delta_{\mathrm{Ct}}}$ method. The RT–qPCR primer sequences are listed in Supplementary Table 2. The average Ct values of the RT–qPCR are listed in Supplementary Table 3.

### Reporting summary

Further information on research design is available in the Nature Portfolio Reporting Summary linked to this article.

## Supplementary information

Supplementary Information Supplementary Figs. 1 and 2, Tables 1–3, Methods and References.

Reporting Summary

## Source data

Source Data Statistical source data for Figs. 1–7 and Extended Data Figs. 1–10.

## Data Availability

The scRNA-seq, RNA-seq of endocardial cells and RNA-seq of untouched ventricles and injured tissues data reported in this study have been deposited in the Gene Expression Omnibus under accession nos. GSE262247, GSE262351 and GSE262169, respectively.
