# Supplementary material for: The innate immune regulator MyD88 dampens fibrosis during zebrafish heart regeneration
Source: Nat Cardiovasc Res. 2024 Sep 13;3(9):1158–76. doi: 10.1038/s44161-024-00538-5 (PMC11399109; doi:10.1038/s44161-024-00538-5)
Supplement: Supplementary file 1 — Supplementary Figs. 1 and 2, Tables 1–3, Methods and References. [file 44161_2024_538_MOESM1_ESM.pdf]

nature cardiovascular research

Supplementary information https://doi.org/10.1038/s44161-024-00538-5

The innate immune regulator MyD88 dampens fibrosis during zebrafish heart regeneration

In the format provided by the authors and unedited

TABLE OF CONTENTS

SUPPLEMENTARY FIGURES 2
SUPPLEMENTARY FIGURE LEGENDS 4
REFERENCES 4
TABLES 5

SUPPLEMENTARY FIGURES

Supplementary Fig. 1

a

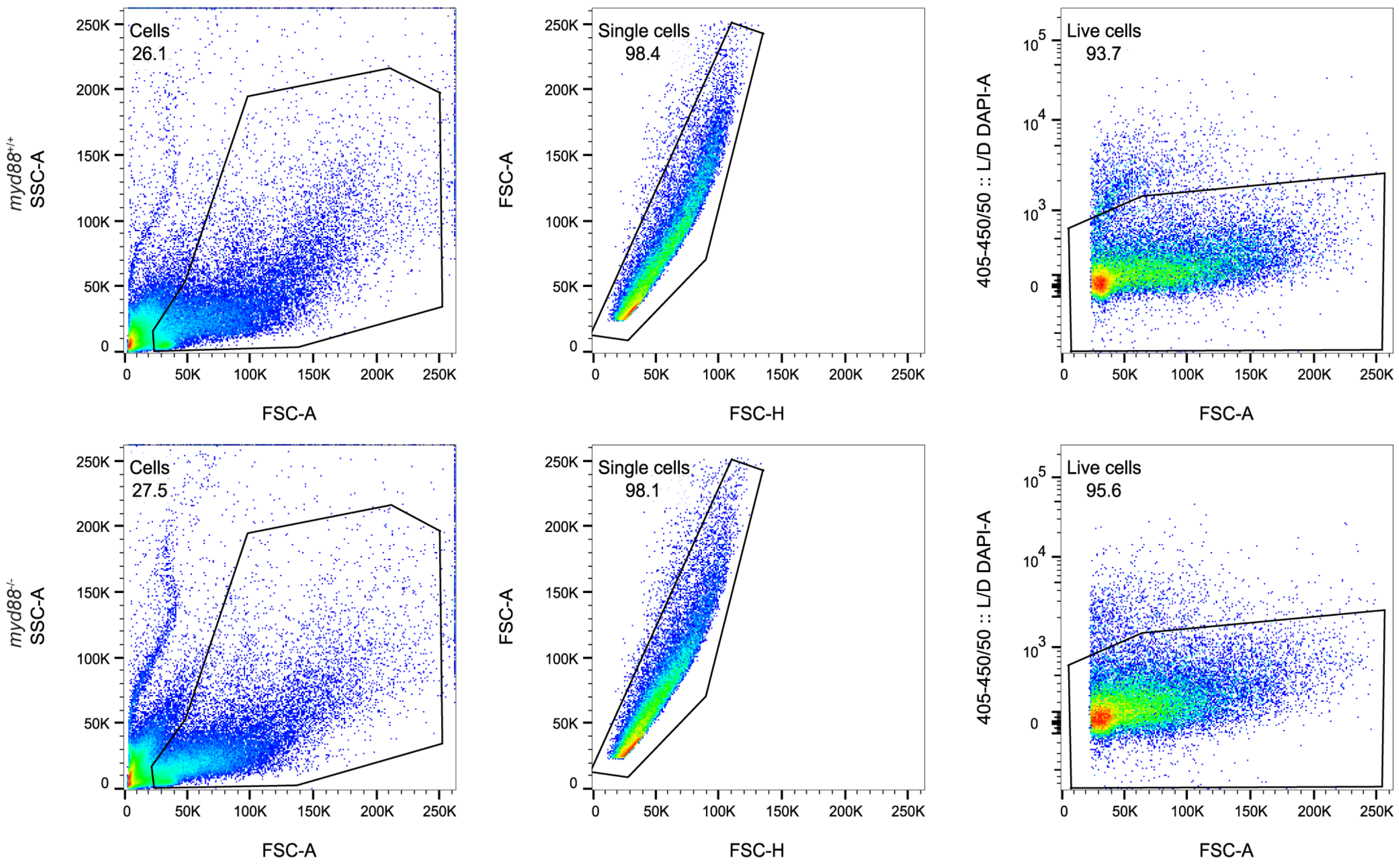

b

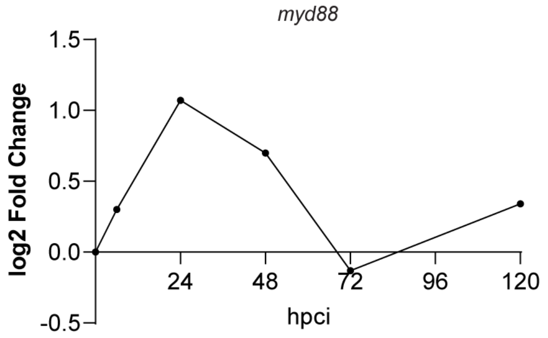

Supplementary Fig. 2

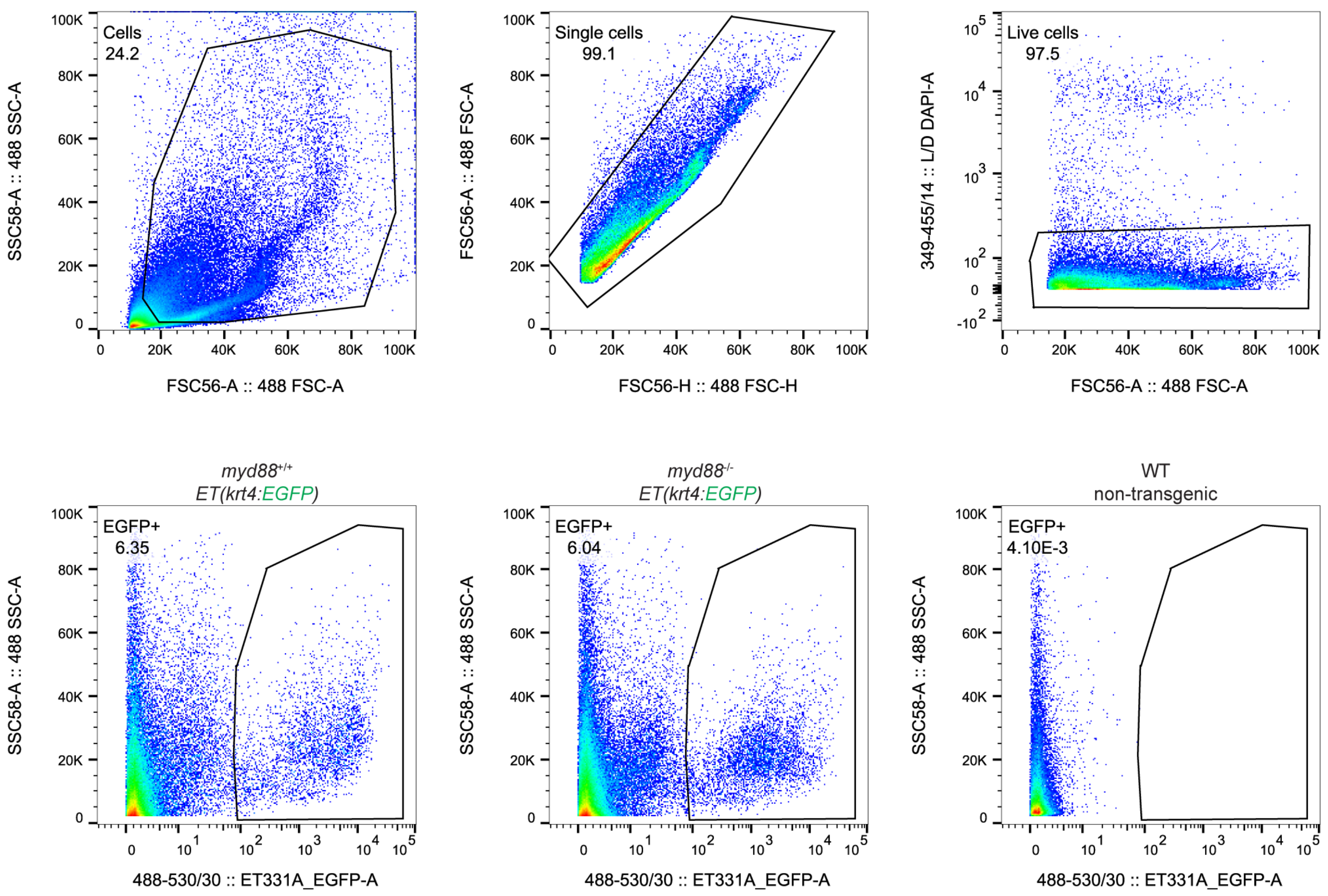

SUPPLEMENTARY FIGURE LEGENDS

Supplementary Fig. 1. Gating strategy for sorting live cells for the scRNA-seq analysis, and *myd88* expression profile during cardiac regeneration.

a, Following exclusion of debris, cell population (Cells) was selected, from which single cells (Single cells) were gated with the FSC-A vs. FSC-H parameters. Within this single cell population, live cells (Live cells) were gated by exclusion of the DAPI^{+} population. Representative plots for sorting live cells from 24 hpci *myd88*$^{+/+}$ and *myd88*$^{-/-}$ ventricles are shown. **b,** *myd88* expression levels in untouched ventricles and in 6, 24, 48, 72 and 120 hpci ventricles. Results analyzed from published bulk RNA sequencing dataset[1].

Supplementary Fig. 2. Gating strategy for sorting endocardial cells for the bulk RNA-seq analysis. Following exclusion of debris, cell population (Cells) was selected, from which single cells (Single cells) were gated with the FSC-A vs. FSC-H parameters. Within this single cell population, live cells (Live cells) were gated by exclusion of the DAPI^{+} population from which endocardial cells (EGFP^{+}) were sorted. Representative plots for sorting live *krt4:*EGFP^{+} cells from cryoinjured *ET(krt4:EGFP); myd88*$^{+/+}$ and *ET(krt4:EGFP); myd88*$^{-/-}$ ventricles at 96 hpci are shown; non-transgenic WT ventricles were used to define the EGFP sorting gate.

REFERENCES

1 Lai, S. L. *et al.* Reciprocal analyses in zebrafish and medaka reveal that harnessing the immune response promotes cardiac regeneration. *Elife* **6**, doi:10.7554/eLife.25605 (2017).

TABLES

Table S1. Partial list of genes downregulated in the *myd88*$^{-/-}$ endocardium at 96 hpci

(genes related to the PI3K/AKT pathway)

Ensembl gene id	Ensembl gene	log2FoldChange	*P* value
ENSDARG00000003776	*pip4k2aa*	-2.36	0.0
ENSDARG00000076554	*cdkn1a*	-1.09	0.0
ENSDARG00000006508	*pip5k1bb*	-1.01	0.0
ENSDARG00000073744	*ip6k1*	-0.97	0.0
ENSDARG00000094673	*rhoab*	-0.90	0.0
ENSDARG00000103038	*pik3r3a*	-0.79	0.0
ENSDARG00000038524	*pik3r1*	-0.75	0.0

Table S2. RT-qPCR primers

	Primer sequence (5'-3')
rpl13a Fw	TCTGGAGGACTGTAAGAGGTATG
rpl13a Rv	AGACGCACAATCTTGAGAGCAG
cxcl18b Fw	TCTTCTGCTGCTGCTTGCGGT
cxcl18b Rv	GGTGTCCCTGCGAGCACGAT
myd88 Fw	GATGATCCACAGGGACTGACACC
myd88 Rv	GGAAGGACGTCTCTGTCAAACAC
mCherry Fw	GAACGGCCACGAGTTCGAGA
mCherry Rv	CTTGGAGCCGTACATGAACTGAGG

Table S3. Average Ct values of RT-qPCRs

Fig. 6c				
	1 hpci		96 hpci	
	myd88$^{+/+}$	*myd88*$^{-/-}$	*myd88*$^{+/+}$	*myd88*$^{-/-}$
rpl13a	23.11	22.51	20.63	20.41
cxcl18b	26.14	26.09	25.73	25.90
Extended Data Fig. 9a				
	no transgene		*Tg(fli1a:myd88,EGFP)*	
	24 hpf larvae	96 hpci ventricles	24 hpf larvae	96 hpci ventricles
rpl13a	16.90	20.39	17.55	20.46
myd88	26.59	26.62	25.32	25.05
Extended Data Fig. 10c				
	Control		*Cre* mRNA injected	
rpl13a	18.91		18.35	
cxcl18b	25.79		20.20	
mCherry	26.08		20.02	
Extended Data Fig. 10f				
	cxcl18b$^{+/+}$		*cxcl18b*$^{-/-}$	
rpl13a	18.69		19.08	
cxcl18b	27.89		34.26	
