# Supplementary material for: The innate immune regulator MyD88 dampens fibrosis during zebrafish heart regeneration
Source: Nat Cardiovasc Res. 2024 Sep 13;3(9):1158–76. doi: 10.1038/s44161-024-00538-5 (PMC11399109; doi:10.1038/s44161-024-00538-5)
Supplement: Supplementary file 2 — Reporting Summary [file 44161_2024_538_MOESM2_ESM.pdf]

nature portfolio

Corresponding author(s): Didier Y. R. Stainier

Last updated by author(s): Aug 2, 2024

# Reporting Summary

Nature Portfolio wishes to improve the reproducibility of the work that we publish. This form provides structure for consistency and transparency in reporting. For further information on Nature Portfolio policies, see our Editorial Policies and the Editorial Policy Checklist.

## Statistics

For all statistical analyses, confirm that the following items are present in the figure legend, table legend, main text, or Methods section.

| n/a | Confirmed | |
|---|---|---|
| ☐ | ☒ | The exact sample size ($n$) for each experimental group/condition, given as a discrete number and unit of measurement |
| ☐ | ☒ | A statement on whether measurements were taken from distinct samples or whether the same sample was measured repeatedly |
| ☐ | ☒ | The statistical test(s) used AND whether they are one- or two-sided<br>*Only common tests should be described solely by name; describe more complex techniques in the Methods section.* |
| ☒ | ☐ | A description of all covariates tested |
| ☐ | ☒ | A description of any assumptions or corrections, such as tests of normality and adjustment for multiple comparisons |
| ☐ | ☒ | A full description of the statistical parameters including central tendency (e.g. means) or other basic estimates (e.g. regression coefficient) AND variation (e.g. standard deviation) or associated estimates of uncertainty (e.g. confidence intervals) |
| ☐ | ☒ | For null hypothesis testing, the test statistic (e.g. $F$, $t$, $r$) with confidence intervals, effect sizes, degrees of freedom and $P$ value noted<br>*Give P values as exact values whenever suitable.* |
| ☒ | ☐ | For Bayesian analysis, information on the choice of priors and Markov chain Monte Carlo settings |
| ☒ | ☐ | For hierarchical and complex designs, identification of the appropriate level for tests and full reporting of outcomes |
| ☒ | ☐ | Estimates of effect sizes (e.g. Cohen's $d$, Pearson's $r$), indicating how they were calculated |

*Our web collection on statistics for biologists contains articles on many of the points above.*

## Software and code

Policy information about availability of computer code

| | |
|---|---|
| Data collection | - Fluorescence images were acquired using ZEN 3.2 (Blue edition) software and the Nikom NIS-AR software (v.5.3).<br>- Wholemount ventricle images were acquired using NIS-Elements 4.30 software.<br>- RT-qPCR data were acquired using Bio-Rad CFX Manager 3.1. |
| Data analysis | Quantifications:<br>- Quantifications of immunostained data were done using the ZEN 3.2 (Blue edition) software.<br>- Quantifications for the coronary vessel coverage and scar area analyses were done using ImageJ (version 1.53c).<br><br>Statistical analysis:<br>- All statistical analyses were performed in GraphPad Prism (v.9).<br>- All graphs were generated using GraphPad Prism (v.9).<br><br>Sequence analysis:<br>- Sequence analysis was performed using ApE Software (v2.0.61).<br><br>Flow cytometry:<br>- Flow cytometry results were analyzed using FlowJo™ (v10.8.1) software from BD Life Sciences.<br><br>scRNA-seq:<br>- Raw reads were aligned against the zebrafish genome (DanRer11) and counted by StarSolo followed by secondary analysis in Annotated Data Format. |

- Preprocessed counts were further analyzed using Scanpy.
- Data visualization was done by CellxGene package (doi:10.5281/zenodo.3235020).

Bulk RNA-seq of untouched ventricles and of injured tissues:
- Sequencing was performed on NextSeq500 instrument (Illumina) using v2 chemistry with 1x75bp single end setup.
- Trimmomatic version 0.39 was employed to trim reads after a quality drop below a mean of Q15 in a window of 5 nucleotides and keeping only filtered reads longer than 15 nucleotides.
- Reads were aligned versus Ensembl zebrafish genome version danRer11 (Ensembl release 99) with STAR 2.7.3a.
- Alignments were filtered to remove duplicates with Picard 2.21.7, multi-mapping, ribosomal, or mitochondrial reads.
- Gene counts were established with featureCounts version 1.6.5.
- The raw count matrix was normalized with DESeq2 version 1.26.0.
- Heatmaps with DEGs were obtained using the Webbased Interactive Omics visualizatioN – Applications (WIlsON).

Bulk RNA-seq of endocardial cells:
- Sequencing was performed on Illumina Sequencing PE150 by the company Novogene.
- The Trimmomatic tool was used to trim reads.
- Reads were aligned versus Ensembl zebrafish genome version danRer11 (Ensembl release 109) with STAR aligner (Version 2.7.10a).
- Alignments were filtered to remove duplicates with Picard tool (version 3.0.0), multi-mapping, ribosomal, and mitochondrial reads.
- Gene counts were generated by the featureCounts version 2.0.4.
- The raw count matrix was normalized and contrasts were created and analyzed by applying the DESeq2 version 1.36.0.
- Heatmaps with DEGs were obtained using the Webbased Interactive Omics visualizatioN – Applications (WIlsON).
- The GSEA analysis python package gseapy (Fang et al, Bioinformatics, 2022) was used.

For manuscripts utilizing custom algorithms or software that are central to the research but not yet described in published literature, software must be made available to editors and reviewers. We strongly encourage code deposition in a community repository (e.g. GitHub). See the Nature Portfolio guidelines for submitting code & software for further information.

## Data

Policy information about availability of data

All manuscripts must include a data availability statement. This statement should provide the following information, where applicable:

- Accession codes, unique identifiers, or web links for publicly available datasets
- A description of any restrictions on data availability
- For clinical datasets or third party data, please ensure that the statement adheres to our policy

The scRNA-seq, RNA-seq of endocardial cells and RNA-seq of untouched ventricles and of injured tissues data reported in the study have been deposited in the Gene Expression Omnibus (GEO) database under accession codes GSE262248, GSE262351 and GSE262169, respectively.

scRNA-seq: Raw reads were aligned against the zebrafish genome (danRer11)
Bulk RNA-seq of untouched ventricles and of injured tissues: Reads were aligned against the zebrafish genome (danRer11 - Ensembl release 99)
Bulk RNA-seq of endocardial cells: Reads were aligned against the zebrafish genome (danRer11, Ensembl release 109)

## Research involving human participants, their data, or biological material

Policy information about studies with human participants or human data. See also policy information about sex, gender (identity/presentation), and sexual orientation and race, ethnicity and racism.

| | |
|---|---|
| Reporting on sex and gender | N/A |
| Reporting on race, ethnicity, or other socially relevant groupings | N/A |
| Population characteristics | N/A |
| Recruitment | N/A |
| Ethics oversight | N/A |

Note that full information on the approval of the study protocol must also be provided in the manuscript.

# Field-specific reporting

Please select the one below that is the best fit for your research. If you are not sure, read the appropriate sections before making your selection.

☒ Life sciences ☐ Behavioural & social sciences ☐ Ecological, evolutionary & environmental sciences

For a reference copy of the document with all sections, see nature.com/documents/nr-reporting-summary-flat.pdf

# Life sciences study design

All studies must disclose on these points even when the disclosure is negative.

| | |
|---|---|
| Sample size | Sample sizes were chosen based on accepted standards in the field using adult animals and previously published literature (such as PMID: 34516874, 26472034 or 29610343). No statistical method was used to predetermine the sample size. |
| Data exclusions | No data were excluded from the analyses. |
| Replication | All experiments in which statistical significance was necessary were verified with at least 3 biological replicates and/or independent experiments. |
| Randomization | All experiments used females and males. For experiments using mutants, the genotype was known prior experiments. All animals were chosen randomly considering the above-described conditions. |
| Blinding | Experiments and image analysis were not performed blindly.<br>- We wanted to know the genotype of the fish used in the study prior to performing experiments, so that we could minimize the use of surplus animals.<br>- Our study builds on previous research where similar methodologies were employed without blinding, and the results were robust and reproducible.<br>- To address potential bias, we ensured that all researchers involved in performing experiments and data analysis underwent thorough training and adhered to standardized protocols. In addition, quantifications were done in a consistent manner (same person, same software) every time and thus even if there is some technical bias, it is the same throughout all the samples (WT and mutant). This consistency helped to maintain the reliability and validity of our findings. |

# Reporting for specific materials, systems and methods

We require information from authors about some types of materials, experimental systems and methods used in many studies. Here, indicate whether each material, system or method listed is relevant to your study. If you are not sure if a list item applies to your research, read the appropriate section before selecting a response.

## Materials & experimental systems

| n/a | Involved in the study | |
|---|---|---|
| ☐ | ☒ | Antibodies |
| ☒ | ☐ | Eukaryotic cell lines |
| ☒ | ☐ | Palaeontology and archaeology |
| ☐ | ☒ | Animals and other organisms |
| ☒ | ☐ | Clinical data |
| ☒ | ☐ | Dual use research of concern |
| ☒ | ☐ | Plants |

## Methods

| n/a | Involved in the study | |
|---|---|---|
| ☒ | ☐ | ChIP-seq |
| ☐ | ☒ | Flow cytometry |
| ☒ | ☐ | MRI-based neuroimaging |

## Antibodies

| | |
|---|---|
| Antibodies used | Primary antibodies used:<br>anti-GFP at 1:500 (chicken, GFP-1010, Aves Labs),<br>anti-αSMA at 1:200 (rabbit, GTX124505, GeneTex),<br>anti-Fli1 [EPR4646] at 1:100 (rabbit, ab133485, Abcam),<br>anti-pAkt [6F5] at 1:200 (mouse, 05-1003, Sigma-Aldrich),<br>anti-PCNA [PC10] at 1:200 (mouse, sc-56, Santa Cruz Biotechnology),<br>anti-Mef2 at 1:100 (rabbit, DZ01398, Boster Bio),<br>anti-Mpx at 1:200 (rabbit, GTX128379, GeneTex),<br>anti-Aldh1a2 [G-2] at 1:100 (mouse, sc-393204, Santa Cruz),<br>anti-Aldh1a2 at 1:200 (rabbit, GTX124302, GeneTex),<br>anti–zf-Cdh5 at 1:100 (rabbit, AS-55715, AnaSpec),<br>anti-pERK [D13.14.4E] at 1:100 (rabbit, 4370S, Cell Signaling Technology),<br>anti-RFP at 1:200 (rabbit, 600-401-379, Rockland),<br>N2.261 at 1:20 (mouse, developed by H. M. Blau, obtained from the Developmental Studies Hybridoma Bank, Iowa City, IA, USA), and<br>anti-DsRed at 1:200 (recognizing mCherry, Living Colors®, rabbit, 632496, Takara).<br><br>Secondary antibodies used:<br>anti-chicken IgG (H+L) Alexa Fluor 488 at 1:500 (goat, A-11039, Invitrogen)<br>anti-mouse IgG (H+L) Alexa Fluor 488 at 1:500 (goat, A-11029, Invitrogen)<br>anti-mouse IgG (H+L) Alexa Fluor 568 at 1:500 (goat, A-11004, Invitrogen)<br>anti-rabbit IgG (H+L) Alexa Fluor 647 at 1:500 (goat, A-21244, Invitrogen) |

Phalloidin-Alexa 568-conjugated at 1:200 (A12380, Thermo Fisher Scientific)

Validation

All antibodies used in this study were commercially available.

anti-GFP, https://www.aveslabs.com/products/anti-green-fluorescent-protein-antibody-gfp.
Manufacturer: Chickens were immunized with purified recombinant green fluorescent protein (GFP) emulsified in Freund's adjuvant.
Reference: PMID: 34732708

anti-αSMA, https://www.genetex.com/Product/Detail/Acta2-antibody/GTX124505
Manufacturer: Reactivity with human and Zebrafish
Reference: PMID: 34516874

anti-Fli1, https://www.abcam.com/products/primary-antibodies/fli1-antibody-epr4646-ab133485.html
Reference: PMID: 29762122

anti-pAkt, https://www.merckmillipore.com/DE/de/product/Anti-phospho-Akt-Ser473-Antibody-clone-6F5,MM_NF-05-1003
Manufacturer: Has been published & validated for use in ELISA, WB, FC, IF, IH
Reference: PMID: 33444612

anti-PCNA, https://www.scbt.com/p/pcna-antibody-pc10
Reference: PMID: 35264012

anti-Mef2, https://www.bosterbio.com/polyclonal-anti-mef2-antibody-dz01398-1-boster.html
Manufacturer: anti-Zebrafish Mef2 Antibody
Reference: PMID: 36513650

anti-Mpx, https://www.genetex.com/Product/Detail/Mpx-antibody/GTX128379
Manufacturer: Mpx antibody detects Mpx protein on zebrafish by whole mount immunohistochemical analysis. Sample: 2 days-post-fertilization zebrafish embryo.
Reference: PMID: 28632131

anti-Aldh1a2, https://www.scbt.com/p/aldh1a2-antibody-g-2
Reference: PMID: 34516874

anti-Aldh1a2, https://www.genetex.com/Product/Detail/Aldh1a2-antibody/GTX124302
Manufacturer: reactivity: zebrafish
Reference: PMID: 36513650

anti-zf-Cdh5
Manufacturer: reactivity: zebrafish. This antibody has been validated by ELISA.
Reference: PMID: 34516874

anti-pERK, https://www.cellsignal.com/products/primary-antibodies/phospho-p44-42-mapk-erk1-2-thr202-tyr204-d13-14-4e-xprabbit-mab/4370
Manufacturer: reactivity with many species, including zebrafish
Reference: PMID: 35285802

anti-RFP, https://www.rockland.com/categories/primary-antibodies/rfp-antibody-pre-adsorbed-600-401-379/
Manufacturer: RFP Antibody Pre-adsorbed from Rockland Immunochemicals is a high-quality, rabbit polyclonal antibody specifically designed for the detection of red fluorescent protein (RFP) and its variants like mCherry, mScarlet, and tdTomato. This product is suitable for a broad range of applications including Western blot (WB), ELISA, immunohistochemistry (IHC), immunofluorescence (IF), flow cytometry (FC), and more. It is uniquely pre-adsorbed to minimize cross-reactivity, offering high specificity and sensitivity. With over 900 references, this product stands as a proven tool in the scientific community, supporting a wide range of research studies from cellular signaling to disease pathology.
Reference: PMID: 35264012

N2.261, https://dshb.biology.uiowa.edu/N2-261
Reference: PMID: 25557620

anti-DsRed, https://www.takarabio.com/documents/Certificate%20of%20Analysis/632496/632496-101717.pdf
Manufacturer: This antibody recognizes DsRed-Express, DsRed-Express2, mCherry, DsRed2, E2-Crimson, tdTomato, mStrawberry, and mBanana, and both N- and C-terminal fusion proteins containing these fluorescent proteins in mammalian cell lysates.

Phalloidin-Alexa 568-conjugated, https://www.thermofisher.com/order/catalog/product/A12380?SID=srch-srp-A12380
Manufacturer: Alexa Fluor 568 phalloidin can be used to visualize and quantitate F-actin in tissue sections, cell cultures, or cell-free preparations.
Reference: PMID: 34516874

## Animals and other research organisms

Policy information about studies involving animals; ARRIVE guidelines recommended for reporting animal research, and Sex and Gender in Research

Laboratory animals

Wild-type, transgenic and mutant zebrafish used in this study (3 to 12 months of age) were from the AB strain. The following mutant

| | |
|---|---|
| Laboratory animals | and transgenic lines were used: myd88 hu3568, cxcl18b bns683, TgBAC(mpx:GFP)i114, Tg(mpeg1:EGFP)gl22, ET(krt4:EGFP)sqet33-1A, Tg(-0.8flt1:RFP)hu5333, Tg(fli1a:myd88,EGFP)bns703, Tg(cxcl18b:EGFP)ibl150, Tg(hsp70l:LoxP-TagBFP-LoxP-cxcl18b-t2a-mCherry)bns660. |
| Wild animals | No wild animals were used in this study. |
| Reporting on sex | All experiments used females and males. Sex was determined based on standard anatomical procedures described for the species. |
| Field-collected samples | No field-collected samples were used in this study. |
| Ethics oversight | All procedures performed on animals conform to the guidelines from Directive 2010/63/EU of the European Parliament on the protection of animals used for scientific purposes and were approved by the Animal Protection Committee (Tierschutzkommission) of the Regierungspräsidium Darmstadt (reference: B2/1218 and B2/1229). |

Note that full information on the approval of the study protocol must also be provided in the manuscript.

## Plants

| | |
|---|---|
| Seed stocks | *Report on the source of all seed stocks or other plant material used. If applicable, state the seed stock centre and catalogue number. If plant specimens were collected from the field, describe the collection location, date and sampling procedures.* |
| Novel plant genotypes | *Describe the methods by which all novel plant genotypes were produced. This includes those generated by transgenic approaches, gene editing, chemical/radiation-based mutagenesis and hybridization. For transgenic lines, describe the transformation method, the number of independent lines analyzed and the generation upon which experiments were performed. For gene-edited lines, describe the editor used, the endogenous sequence targeted for editing, the targeting guide RNA sequence (if applicable) and how the editor was applied.* |
| Authentication | *Describe any authentication procedures for each seed stock used or novel genotype generated. Describe any experiments used to assess the effect of a mutation and, where applicable, how potential secondary effects (e.g. second site T-DNA insertions, mosiacism, off-target gene editing) were examined.* |

## Flow Cytometry

### Plots

Confirm that:

☒ The axis labels state the marker and fluorochrome used (e.g. CD4-FITC).

☒ The axis scales are clearly visible. Include numbers along axes only for bottom left plot of group (a 'group' is an analysis of identical markers).

☒ All plots are contour plots with outliers or pseudocolor plots.

☒ A numerical value for number of cells or percentage (with statistics) is provided.

### Methodology

| | |
|---|---|
| Sample preparation | For the scRNA-seq experiment, cardiac cells were isolated from a pool of 4 myd88+/+ and 4 myd88-/- ventricles, and for the endocardial bulk RNA sequencing experiment from a pool of 4 ET(krt4:EGFP); myd88+/+ and 4 ET(krt4:EGFP); myd88-/- ventricles for each sample. Cell isolation was performed following the manufacturer's instructions (Pierce Primary Cardiomyocyte Isolation Kit, Thermo Fisher Scientific, Cat#88281) and with the following modifications: Incubation was performed at 30°C for 20 min, followed by resuspension in 1× Hanks' balanced salt solution (Gibco, Cat# 14175053) with 0.25% BSA. Cell suspension was passed through a round bottom polystyrene test tube fitted with 35 µm nylon mesh filter cap (Falcon® #352235, Corning Inc.). DAPI (Sigma, Cat# D954) was added prior to sorting. For the scRNA-seq experiment, resuspended cells were sorted using a BD FACSAria™ III Cell Sorter (BD Biosciences), equipped with 100µm nozzle and at instrument pressure setting of 20 psi. Dead cells were excluded using DAPI excited by a 30 mW 405 nm laser paired with 450/50 nm band pass filter. For the endocardial bulk RNA sequencing experiment, resuspended cells were sorted using an Invitrogen Bigfoot Spectral cell sorter (ThermoFisher Scientific) equipped with 100µm nozzle tip and at instrument pressure setting of 20 psi. Dead cells were excluded using DAPI excited by a 100 mW 355 nm laser paired with 455/14 nm band pass filter. EGFP fluorescence was measured with 100 mW 488 nm excitation paired with 530/30 band pass filter. Sorted EGFP+ cells were resuspended in ice-cold QIAzol Lysis Reagent (Qiagen), flash-frozen in liquid nitrogen and kept at -80ºC until RNA extraction. Cytometric data were recorded using FACSDiva software (Version 8.0.1; BD Biosciences), and Sasquatch Software (Version 1.19.2; ThermoFisher Scientific). |
| Instrument | BD FACSAria™ III Cell Sorter (BD Biosciences) and Bigfoot Spectral cell sorter (ThermoFisher Scientific) |
| Software | FlowJo™ v10.8.1 software (BD Life Sciences) |
| Cell population abundance | Sample purity was determined by visualizing the single cell suspension under a microscope. The sorted EGFP+ cells from the ET(krt4:EGFP)sqet33-1A line were further visualized in the EGFP channel alongside the negative cell populations in a fluorescent microscope. Cell abundance differed as per the transgenic lines used and the chosen time-point following cardiac cryoinjury. |
| Gating strategy | Debris was excluded (SSC-A/FSC-A), followed by doublet exclusion (FSC-A/FSC-H). Within this single cell population, live cells |

Gating strategy

were gated by exclusion of DAPI+ population (DAPI-A/FSC-A).
For sorting endocardial cells from the ET(krt4:EGFP)sqet33-1A line, EGFP+ cells were further sorted and collected (SSC-A/EGFP-A). Non-transgenic WT ventricles used to define the EGFP sorting gate.

☒ Tick this box to confirm that a figure exemplifying the gating strategy is provided in the Supplementary Information.
